# Templated Pluripotent Stem Cell Differentiation via Substratum-Guided Artificial Signaling

**DOI:** 10.1021/acsbiomaterials.4c00885

**Published:** 2024-10-01

**Authors:** Hannah J. Brien, Joanne C. Lee, Jhanvi Sharma, Catherine A. Hamann, Madeline R. Spetz, Ethan S. Lippmann, Jonathan M. Brunger

**Affiliations:** † Department of Biomedical Engineering, Vanderbilt University, Nashville, Tennessee 37235, United States; ‡ Department of Chemical and Biomolecular Engineering, Vanderbilt University, Nashville, Tennessee 37235, United States; § Center for Stem Cell Biology, Vanderbilt University, Nashville, Tennessee 37235, United States

**Keywords:** synthetic biology, surface engineering, pluripotent stem cells, synNotch, morphogenesis

## Abstract

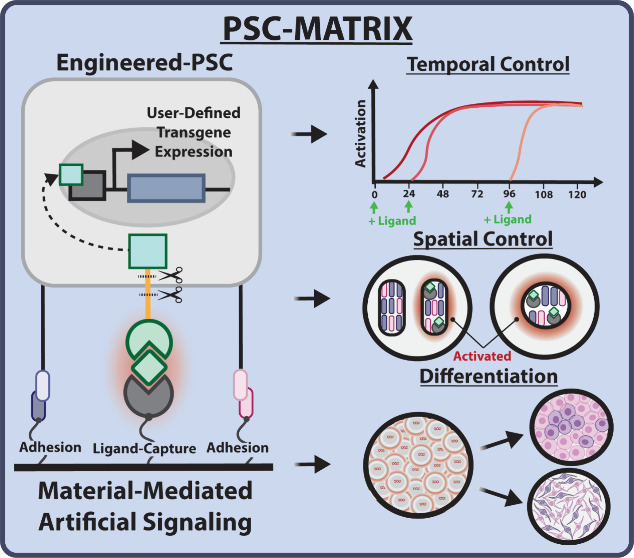

The emerging field of synthetic morphogenesis implements synthetic biology tools to investigate the minimal cellular processes sufficient for orchestrating key developmental events. As the field continues to grow, there is a need for new tools that enable scientists to uncover nuances in the molecular mechanisms driving cell fate patterning that emerge during morphogenesis. Here, we present a platform that combines cell engineering with biomaterial design to potentiate artificial signaling in pluripotent stem cells (PSCs). This platform, referred to as PSC-MATRIX, extends the use of programmable biomaterials to PSCs competent to activate morphogen production through orthogonal signaling, giving rise to the opportunity to probe developmental events by initiating morphogenetic programs in a spatially constrained manner through non-native signaling channels. We show that the PSC-MATRIX platform enables temporal and spatial control of transgene expression in response to bulk, soluble inputs in synthetic Notch (synNotch)-engineered human PSCs for an extended culture of up to 11 days. Furthermore, we used PSC-MATRIX to regulate multiple differentiation events via material-mediated artificial signaling in engineered PSCs using the orthogonal ligand green fluorescent protein, highlighting the potential of this platform for probing and guiding fate acquisition. Overall, this platform offers a synthetic approach to interrogate the molecular mechanisms driving PSC differentiation that could be applied to a variety of differentiation protocols.

## Introduction

Pluripotent stem cells (PSCs) are unique cells with the capability to self-renew and differentiate into any terminal state.^1−3^ The proliferation and differentiation of stem cells are controlled by their local, dynamic microenvironment through cell-cell and cell-extracellular matrix interactions as well as chemical and mechanical stimuli that influence adhesion, motility, signal transduction, gene expression, and morphology.^3−9^ Thus, under appropriately defined conditions, one could theoretically orchestrate the development of arbitrarily selected tissues or organs from a single pluripotent stem cell, and this prospect makes such a cell a promising tool for developmental biologists and tissue engineers. However, the field is currently limited by the lack of fundamental knowledge on the complex interplay of biochemical and biophysical factors that determine human stem cell fate allocation in hierarchical tissues.^4,9−11^ During embryogenesis, PSCs communicate with each other to self-organize and differentiate through a variety of complex mechanisms, including those that fit reaction-diffusion (RD), positional information (PI), and lateral inhibition models of morphogenesis.^12−14^ While significant strides toward understanding morphogenesis have been made in studies that investigated the interactive RD and PI mechanisms involved in PSC differentiation, most include bulk supplementation of recombinant factors or chemical surrogates in the cell culture medium, representing a bottleneck to our understanding of cell-mediated events that drive fate acquisition.^15−18^ In an effort to overcome this challenge, several synthetic biology approaches have been refined to specify patterning events reminiscent of morphogenetic programs in response to chemical, mechanical, or optical inputs.^19−25^ These synthetic approaches offer a rational method to design, build, and test genetic circuits based on the current understanding of morphogenetic systems. Following this process with an analysis of resultant morphogenetic outcomes allows the field to uncover nuances in the molecular mechanisms driving PSC differentiation.^26,27^ However, most of these synthetic morphogenesis gene circuits have been used in cell lines other than PSCs, such as human embryonic kidney cells (HEK-293),^19,21,28,29^ restricting our ability to directly explore how artificial gene circuits can guide stem cell organization and lineage commitment.

**Figure 1:**
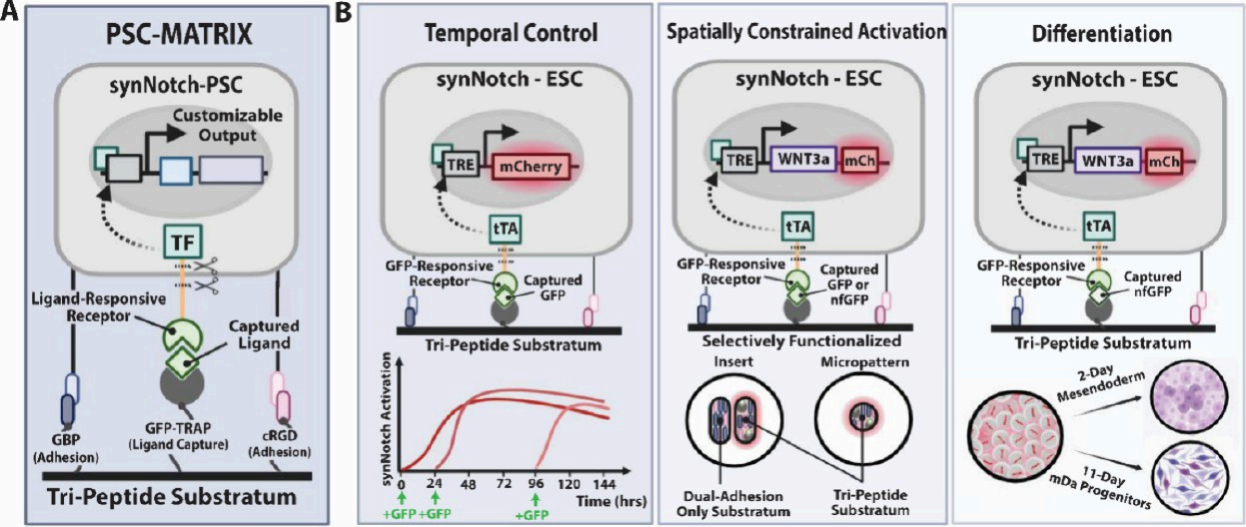
**PSC-MATRIX design and summary of properties of PSC-MATRIX investigated.** (A) Schematic of the PSC-MATRIX platform. The defined substratum is composed of adhesion peptides derived from vitronectin (GAG Binding Peptide, GBP) and fibronectin (cyclic RGD, cRGD) in addition to the GFP-affinity motif (GFP-TRAP). This customized substratum supports PSC adhesion and facilitates the conversion of a soluble orthogonal ligand, GFP, into an immobilized input competent to activate downstream transgene expression via synNotch signaling. (B) PSC-MATRIX was used with mCherry reporter synNotch-hESCs with activating ligand, GFP, dosed at different time points to demonstrate temporal control of artificial signaling events. Then, PSC-MATRIX was used to selectively functionalize regions of a cell culture plate to enable the activation of synNotch-hESCs in a spatially constrained manner. PSC-MATRIX was also implemented to guide mesendoderm/early peri-gastrulation differentiation and to produce midbrain dopaminergic (mDa) neuron progenitors with WNT transgene expression via artificial signaling by the orthogonal ligand nonfluorescent GFP. Figure created with BioRender.

To implement a synthetic biology approach in a PSC-based system, here we engineered human embryonic stem cells (hESCs) and induced pluripotent stem cells (iPSCs) with the modular synthetic Notch (synNotch) receptor platform.^30^ SynNotch is based on the native juxtacrine Notch/Delta signaling channel that requires receptor engagement with an immobilized ligand for signal transduction.^30^ With synNotch (Figure 1A), a user-defined recognition domain triggers combined ectodomain and intramembrane cleavage of the receptor in the presence of an immobilized activating ligand.^30^ After cleavage occurs, the intracellular domain, a transcription factor, is released to regulate transcription of target transgenes. Crucially, ligands must be immobilized for potent synNotch activation; soluble, monomeric ligands do not activate synNotch receptors.^31^ Because the synNotch ligand must be immobilized, synNotch serves as a juxtacrine signaling channel that confers spatially gated, localized cellular responses to selected inputs.^32^ Suggesting the utility of this spatially responsive system, synNotch has been used to program artificial morphogen systems in L929 fibroblasts such that orthogonal cell-cell interactions produce mCherry and green fluorescent protein (GFP) gradients that mirror the positional distribution of morphogens seen *in vivo*.^25^ This and other proof-of-concept studies^33−35^ indicate the feasibility of using synthetic development platforms, such as synNotch, to reconstitute morphogenetic programs.

We recently described a platform called MATRIX (Material Activated To Regulate Inducible gene eXpression), which interfaces synNotch-engineered cells, including PSCs, with programmable biomaterial surfaces that capture and immobilize soluble synNotch ligands.^36^ The biomaterial-anchored ligand is then presented to synNotch cells in a manner that mimics juxtacrine Notch-ligand interactions, giving rise to transgene expression across a wide dynamic range and capable of patterning cellular responses based on biomaterial functionalization with synNotch ligand-capturing motifs. Thus, the MATRIX platform enables spatially regulated transgene expression in response to bulk, soluble inputs, including non-native, bioinert factors such as GFP.^36^ In this initial work, we decorated culture surfaces with adhesion molecules supporting PSC attachment and with the ligand-capturing motif, GFP-TRAP, to regulate inducible expression of Neurogenin-2 (NGN-2), a master transcription factor for converting PSCs to motor neurons, upon GFP activation of synNotch. This iteration of MATRIX was able to orthogonally drive transcription factor-directed differentiation of PSCs toward an early neuronal state after 4 days; however, it was not used with cell-secreted morphogens, nor was it demonstrated to be competent to support the extended culture periods associated with many PSC-based differentiation protocols. Thus, our original description of the MATRIX platform did not demonstrate utility in the area of synthetic morphogenesis, which typically involves prolonged cell culture and relies on cell nonautonomous signaling that features a suite of mechanisms, including those from the aforementioned lateral inhibition, RD, and PI models.^12−14^

Motivated to extend the MATRIX platform to such applications, we sought to determine the key factors that support durable stem cell adhesion and synNotch-driven morphogen expression sufficiently potent and sustained to drive differentiation. In this work, we assess the individual and combined contributions of the stem cell adhesion molecules and the ligand-capturing motif, GFP-TRAP, to PSC adhesion and synNotch activation in high-density cultures often required by differentiation protocols^15,17,37−39^ (Figure 1). Furthermore, we aimed to demonstrate that PSC-MATRIX enables temporal control of artificial signaling events based on when soluble activating ligand is supplemented in the culture medium (Figure 1B). We also present methods for effectively deploying the modified PSC-MATRIX platform in synthetic morphogenesis studies involving both hESCs and hiPSCs in monolayer as well as in spatially constrained, patterned culture (Figure 1B). We then implement the platform to guide mesendoderm/early peri-gastrulation differentiation and to produce midbrain dopaminergic neuron progenitors via WNT transgene expression in response to artificial signaling mediated by the orthogonal ligand GFP (Figure 1B). Our results refine the MATRIX platform for extensive use with PSCs, opening the possibility to probe developmental events via spatially patterned surfaces that can activate morphogenetic programs in defined locations in response to orthogonal signaling channels.

## Materials and Methods

### Engineered Surface Functionalization

To generate the peptide-presenting surfaces, we used combinations of biotinylated adhesion peptides—glycosaminoglycan binding peptide (GBP) (GenScript Express, biotin-Ahx-GKKQRFRHRNRKG), cyclic RGD (cRGD) (Carbosynth, cyclo[Arg-Gly-Asp-d-Phe-Lys(Biotin-PEG-PEG)), and a GFP capturing motif—GFP-TRAP^40,41^ which was conjugated to a biotinylated polyethylene glycol (PEG) linker (PEG GFP-TRAP). PEG GFP-TRAP was prepared as described in Lee et al.^36^ Briefly, GFP-TRAP (Chromotek, #gt-250) was conjugated to a 12-unit PEG linker containing an activated *N*-hydroxysuccinimide ester (NHS) group and biotin group (NHS-PEG-Biotin) (Thermo Scientific, #A35389). GFP-TRAP was reacted with 2-fold molar excess of NHS-PEG-Biotin for 1 h at room temperature and quenched with 50 mM glycine (pH 2.1) to obtain a 7.19 μM stock solution of PEG GFP-TRAP. The adhesion peptides were reconstituted in UltraPure distilled water (Invitrogen, 10977–015) to final concentrations of 10 μM GBP and 17.85 μM cRGD. The adhesion peptides were then diluted 1:1000 in PBS to obtain working stocks for experiments.

### Defined Substratum Preparation

For single peptide surface preparation, the working stock peptides were diluted in DPBS to 5 μM GBP, 5 μM cRGD or 0.4 μM PEG GFP-TRAP. For dual peptide surface preparation, GBP or cRGD was mixed with PEG GFP-TRAP and diluted with DPBS to obtain a final concentration of 5 μM GBP or cRGD and 0.4 μM PEG GFP-TRAP. For the triple peptide surface experiments, GBP, cRGD, and PEG GFP-TRAP were mixed and diluted in PBS to obtain a final concentration of 5 μM GBP, 2.15 μM cRGD, and 0.4 μM PEG GFP-TRAP unless otherwise noted.

To prepare the well plate for surface functionalization, non-tissue culture treated well plates were coated with 10 μg/mL of streptavidin (Thermo Scientific, #21125) in DPBS and incubated at 37 °C overnight. While the streptavidin was adsorbing, the defined substratum was prepared (see above). The streptavidin was then removed and immediately replaced with the defined substratum solution and incubated for 1–2 h at 37 °C. Cells were dissociated into a single-cell suspension using Accutase and plated into the coated wells at a high density of 1.25 × 10^5^ cells/cm^2^ in medium supplemented with 10 μM Y-27632 ROCK inhibitor. Immediately before plating the cells, the peptide solution was removed and replaced with medium.

The Geltrex (Thermo Scientific, #A1413302) and PEG GFP-TRAP surface was prepared by first diluting 50 μL of Geltrex with 12 mL of DMEM/F12 + Glutamax (Gibco, #10565-042). Then, stock PEG GFP-TRAP (7.19 μM) was added to the diluted Geltrex solution to obtain a final PEG GFP-TRAP concentration of 0.8 μM. The mixture was then plated on a non-tissue culture treated plate that had been incubated at 37 °C overnight with 10 μg/mL of streptavidin. The Geltrex + GFP-TRAP solution was incubated for 1–2 h at 37 °C before it was removed and cells were plated in the same conditions as those plated on the defined substratum.

### Plasmid Design and Construction

All plasmids were designed using SnapGene software and assembled using the NEBuilder HiFi DNA assembly mix. SynNotch platform elements were cloned into the *Sleeping Beauty* transposon plasmid (a modified form of Addgene 60495, a kind gift from Eric Kowarz).^42^ Assembled plasmids were transformed into SMOBio champion DH5α *E. coli* (Stellar Scientific, #SMOB-20–200) competent cells and plated on agar plates supplemented with ampicillin. The transformed bacteria were incubated overnight (12–16 h) at 37 °C before colonies were picked and screened via PCR for correct fragment assembly. Positive colonies were grown overnight at 37 °C in LB broth supplemented with ampicillin prior to miniprep DNA purification (Qiagen). All plasmid sequences were verified with Sanger sequencing.

### Establishing synNotch-PSC Lines

H9 hESCs as well as the CC3 and KOLF2.1J iPSCs^43−45^ were engineered to stably express synNotch platform elements (i.e., receptor and transgene payloads) via a *Sleeping Beauty* transposase/transposon system. Using the TransIT-LT1 Transfection Reagent (Mirus, #MIR 2300), 1 μg of the *Sleeping Beauty* 100x transposase (Addgene 34879, a kind gift from Zsuzsanna Izsvak)^46^ and 1 μg of the transposon containing the synNotch receptor and transgene payloads were transfected into hESCS and iPSCs. The WNT3a transgene was a murine coding sequence that is highly conserved and active in human cells. Approximately 1 × 10^6^ cells/mL were plated into a Geltrex-coated well in a 6-well plate and allowed to incubate with the DNA complexes for 24 h at 37 °C in mTeSR Plus medium supplemented with 10 μM Y-27632 rho-associated protein kinase (ROCK) inhibitor (Tocris, #1254). After 24 h, medium was replaced with fresh mTeSR Plus. Engineered cells were selected with 0.6 μg/mL puromycin during subcultivation and sorted at the Vanderbilt Flow Cytometry Shared Resource with a 4-laser FACSAria lll based on receptor expression using a c-myc-tag epitope appended to the synNotch receptor.

### Activation of synNotch Engineered hPSCs

To activate the GFP-responsive, LaG16 synNotch hPSCs, we supplemented culture media with 5 nM GFP or 5 nM of the Y67F variant of GFP, referred to as nonfluorescent GFP (nfGFP). The Y67F variant of GFP preserves the epitopes recognized by GFP-TRAP and the LaG16-synNotch but abolishes the fluorescence of the protein.^47^ GFP or nfGFP was supplemented in the media when cells were plated and with each daily medium change unless otherwise specified.

### Human Embryonic Stem Cells (H9s) and Induced Pluripotent Stem Cells (CC3s and KOLF2.1Js)

H9, CC3 and KOLF2.1J^43,43,45^ stem cells were maintained in mTeSR Plus (Stem Cell Technologies) or Essential 8 (E8) medium^44^ in Geltrex (Gibco)-coated wells. Differentiation experiments were performed in either N2B27 or HUESM base medium, as noted below. Routine cell passages were carried out using ReLeSR (Stem Cell Technologies) to detach cells. For substratum experiments and flow cytometry, cells were dissociated into a single-cell suspension using Accutase (Gibco).

### Media Preparation

Essential 4 (E4) basal medium^48^ was prepared in large batches as a precursor for Essential 6 (E6) and Essential 8 (E8) media by mixing l-Ascorbic acid 2-phosphate sesquimagnesium salt hydrate, sodium selenite solution, and sodium bicarbonate in DMEM/F12, HEPES (Thermo Fisher Scientific, #11330057). E6 medium was prepared by adding 100 μL of human insulin solution (Sigma, #I9278) and 500 μL of 10 mg/mL Transferrin (R&D systems, #2914-HT) to 500 mL of E4 medium. E8 medium was prepared by adding 500 μL of 0.1 mg/mL FGF2 (Peprotech, #100–18B), and 500 μL of 2 μg/mL TGFβ1 (Peprotech, #100–21) to 500 mL of E6 medium. Both E6 and E8 media were sterile filtered prior to cell culture use.

N2B27 medium was prepared by mixing 23.6 mL of DMEM/F12 + Glutamax, 24 mL neurobasal medium (Gibco, #21103–049), 250 μL of MEM NEAA (Gibco, #11140–050), 250 μL of sodium pyruvate (Gibco, #11360–070), 250 μL of Glutamax (Gibco, #35050–061), 250 μL of N2 (Gibco, #17502–048), 500 μL of B27 supplement without vitamin A (Gibco, #12587010), 33.2 μL of 7.5% BSA Fraction V (Gibco, #15260–037), and 225 μL of β-Mercaptoethanol (Gibco, #21985–023).

HUESM medium^37^ was prepared by mixing 38.4 mL of DMEM + Glutamax (Fisher Scientific, #10569–010), 10 mL of KnockOut serum replacement (Gibco, #10828–028), 0.5 mL of MEM NEAA (Gibco, #11140–050), 0.1 mL of β-Mercaptoethanol (Gibco, #21985–023), and 1 mL of B27 supplement without vitamin A (Gibco, #12587010). HUESM medium was conditioned by mouse embryonic fibroblasts (MEFs) (EmbryoMax, Lot: 3050344) prior to use in experiments. For conditioning, MEFs were plated near confluency in 6-well plates coated with 0.1% gelatin (Sigma, #G1393) and incubated overnight. The next day, the medium was removed and replaced with 2 mL of HUESM medium and incubated overnight to condition the medium. After 24 h, medium was collected using a syringe and sterile filtered to obtain HUESM conditioned medium (HUESM-CM). MEFs were used to condition HUESM medium by repeating this process for up to 10 days. After medium was conditioned, it was stored at −20 °C until it was used for experiments.

The base mDa neuron medium^38^ was prepared by mixing 48 mL of neurobasal media, 0.5 mL of Glutamax, 0.5 mL of N2, and 1 mL of B27 supplement minus vitamin A.

### Spatially Constrained PSC Culture

For micropatterned cell culture, a 0.2 μL droplet of 10 μg/mL of streptavidin was added to individual wells of a non-tissue culture treated 96-well plate and incubated at 37 °C for approximately 1 h, or until the streptavidin appeared dried in the well. The wells were then washed with 1% BSA in PBS to minimize nonspecific binding of our biotinylated peptides. The wells were then rinsed with PBS prior to treating the entire well with 5 μM GBP, 2.15 μM cRGD, and 0.4 μM GFP-TRAP to functionalize the droplet. After an hour-long incubation, the excess peptide solution was removed and a 5 μL droplet of approximately 20,000 WNT3a synNotch-hESCs was added to the well on top of the droplet location. The cells were incubated for 30 min prior to supplementing 150 μL of HUESM-CM medium^37^ with or without 5 nM GFP to obtain a confluent micropatterned disc of cells. Medium was not replaced during the 2-day experiment.

For the spatially constrained functionalization experiment, a 2-panel Ibidi insert (Cat # 80209) was placed into a non-tissue culture treated 24-well plate using sterile tweezers. The left panel of the insert was functionalized with only 5 μM GBP and 2.15 μM cRGD and the right panel was functionalized with 5 μM GBP, 2.15 μM cRGD, and 0.4 μM GFP-TRAP. WNT3a synNotch-hESCs were plated in the functionalized panels at the same cell density as the mDa neuron differentiation, 400,000 cells/cm^2^, based on the coating area of the individual panel. After 2 h of incubation, the inserts were removed using sterile tweezers, and the culture medium containing nfGFP was exchanged each day as described below in the mDa neuron differentiation.

### Mesendoderm/Early Peri-gastrulation Differentiation of synNotch H9s

For mesendoderm/peri-gastrulation differentiation, we adapted the protocol described by Deglincerti et al.^37^ Briefly, WNT3a synNotch engineered H9s were dissociated into a single cell suspension using Accutase and resuspended in HUESM-CM supplemented with 10 μM Y-27672 ROCK inhibitor and 20 ng/mL bFGF. Cells were plated at a high density of 1.25 × 10^5^ cells/cm^2^ into a 96-well plate coated with the defined tri-peptide substratum. A subset of the wells was supplemented with 5 nM nfGFP to activate synNotch and induce morphogen expression. Medium was replaced 24 h after plating. After 48 h, the cells were prepared for immunofluorescence as described below.

### mDa Neuron Differentiation of synNotch H9s

For mDa neuron differentiation, we adapted the protocol described in Kim et al.^38^ WNT3a synNotch-H9s or wild-type (WT) H9s were dissociated into a single-cell suspension using Accutase. The WNT3a synNotch-hESCs were plated at a density of 400,000 cells/cm^2^ on the optimized defined substratum on day 0 in base mDa neuron medium supplemented with 250 nM LDN193189 dihydrochloride (LDN) (Tocris #605310), 10 μM SB431542 (SB) (StemCell Technologies #72234), 250 nM Smoothened Agonist (SAG) (Fisher Scientific #43–661), 2 μM Purmorphamine (PM) (Fisher Scientific, #45–511–0), 10 μM Y-27672 ROCK inhibitor, and 0 or 5 nM nfGFP. Culture media were changed every day as outlined in the following: 24 h after plating, the Y-27672 ROCK inhibitor concentration was dropped to 5 μM for the remainder of the differentiation. On day 7, the small molecules LDN, SB, PM, and SAG were withheld. On day 8 and 9, cells were maintained in medium composed of 50% base mDa medium and 50% E6 medium supplemented with 0.4 μM LDN. On day 10, cells were maintained in medium composed of 25% mDa base medium and 75% E6 supplemented with 0.4 μM LDN. WT H9 hESCs were treated identically, with the exception that cells were plated on Geltrex rather than the tri-peptide substratum, and cells received CHIR99021 (CHIR) supplementation of 0.7 μM on days 0–4, 7.5 μM on days 4–10, and 3 μM on day 11 as previously published.^38^ On day 11, cells were prepared for immunofluorescence as described below.

### Measurements

#### Flow Cytometry/FACS

Prior to cell sorting or analytical flow cytometry, cells were dissociated into a single cell suspension using Accutase (Gibco). Cells were spun down at 300*g* and resuspended in 150 μL blocking buffer (5% FBS in DPBS) and placed on ice for 15 min prior to immunolabeling. Cells were stained with an Alexa Fluor 647 conjugated to a mouse monoclonal antibody for c-myc-tag (Cell Signaling Technologies #2233S) diluted 1:50 in blocking buffer. Cells were incubated for an hour before being washed twice with 175 μL of blocking buffer. In preparation for analytical flow cytometry, cells were collected via centrifugation and resuspended in 70 μL of blocking buffer for analysis on a Cellstream flow cytometer. Results were analyzed in FlowJo to obtain mean fluorescence intensity values of singlet synNotch cells. For cell sorting, the same preparation was used, but samples were resuspended at a density of 1 × 10^6^ cells/ml and then run on a 4-laser FACSAria lll cell sorter to collect a population of cells positive for the synNotch receptor. Collected cells were spun down at 300*g* and resuspended in mTeSR Plus +10 μM Y-27632 ROCK inhibitor (Tocris, #1254) and plated in Geltrex-coated well-plates.

#### Microscopy

All images were obtained using a Leica DMi8 epifluorescence microscope. Surface characterization experiments were imaged at 10× magnification. Differentiation experiments were imaged at 20× magnification. For the 2-panel insert experiments, LAS X software was used to stitch and merge individual images. The LAS X software was also used to measure the diameter of individual discs.

#### ImageJ Analysis

Mean pixel intensity was calculated using ImageJ/Fiji software (available from the NIH at https://imagej.net/software/fiji/downloads) using built-in functions.^49^ Briefly, fluorescence images were converted to 8-bit greyscale images and the mean gray value was measured. For mean pixel intensity per nucleus, the DAPI channel image was used to develop a region of interest (ROI) that was used to measure the mean gray value of corresponding fluorescence images. To make the ROI, a DAPI image was converted to binary, holes in the binary image were filled to mark individual nuclei, and particles were analyzed using the default ImageJ settings to count the number of nuclei and save the ROI to the manager. The corresponding fluorescence images were individually converted to greyscale and the ROI was added as an overlay to the image from the manager. Then the mean gray value was calculated for each nucleus (based on the ROI) and then averaged to obtain the mean pixel intensity per nucleus.

#### Immunofluorescence

Cells were fixed in 4% paraformaldehyde in DPBS for 15 min at room temperature. Fixed cells were washed with DPBS prior to being blocked and permeabilized in buffer (5% FBS and 0.3% Triton X in DPBS) for 30 min at room temperature. Cells were incubated with primary antibody, diluted in buffer, for 1 h at room temperature or overnight at 4 °C. Antibody dilutions and sources are shown in Supplemental Table 1. For pluripotency staining, cells were stained for stage-specific embryonic antigen-4 (SSEA 4) and TRA-1–81 (Podocalyxin), two conventional pluripotency markers. For mesendoderm/early peri-gastrulation experiments, cells were stained for the mesendodermal/primitive streak marker Brachyury, the mesendodermal marker VEGFR2, the pluripotency/ectodermal marker SOX2, and the endodermal marker SOX17.^50−52^ For mDa Neuron progenitor experiments, cells were stained for the mDa neuron progenitor markers LMX1a, FOXA2, OTX2, and EN1.^38,53,54^ After incubating cells with primary antibodies, cells were washed twice with DPBS before incubation with secondary antibodies and the nuclear stain DAPI. Cells were protected from light and incubated for 1 h at room temperature. Cells were then washed with DPBS twice prior to imaging cells on a Leica DMi8 microscope. All antibodies were diluted in blocking buffer.

### Statistical Analysis

Statistical analysis was performed in GraphPad Prism version 10.2.2. Bar graphs display the mean of triplicate samples, unless otherwise noted, with error bars showing standard error of the mean (SEM). Statistical significance for comparisons with more than two experimental groups was determined using one-way or two-way ANOVA, as appropriate, followed by a Tukey’s multiple comparisons posthoc test with alpha set to 0.05. For the time-varying GFP-treatment experiment, a repeated measures ANOVA was performed. The within-subjects factor was the different GFP treatment groups (0, 24, 96 h), and the fluorescence measures for days 0–10 were the repeated measures. This was followed by a Tukey’s multiple comparisons posthoc test with alpha set to 0.05. Student’s *t* tests were performed for experimental groups composed of only two experimental conditions with alpha set to 0.05.

## Results

### An Engineered Substratum Supports PSC Adhesion and Potentiates synNotch Signaling

**Figure 2:**
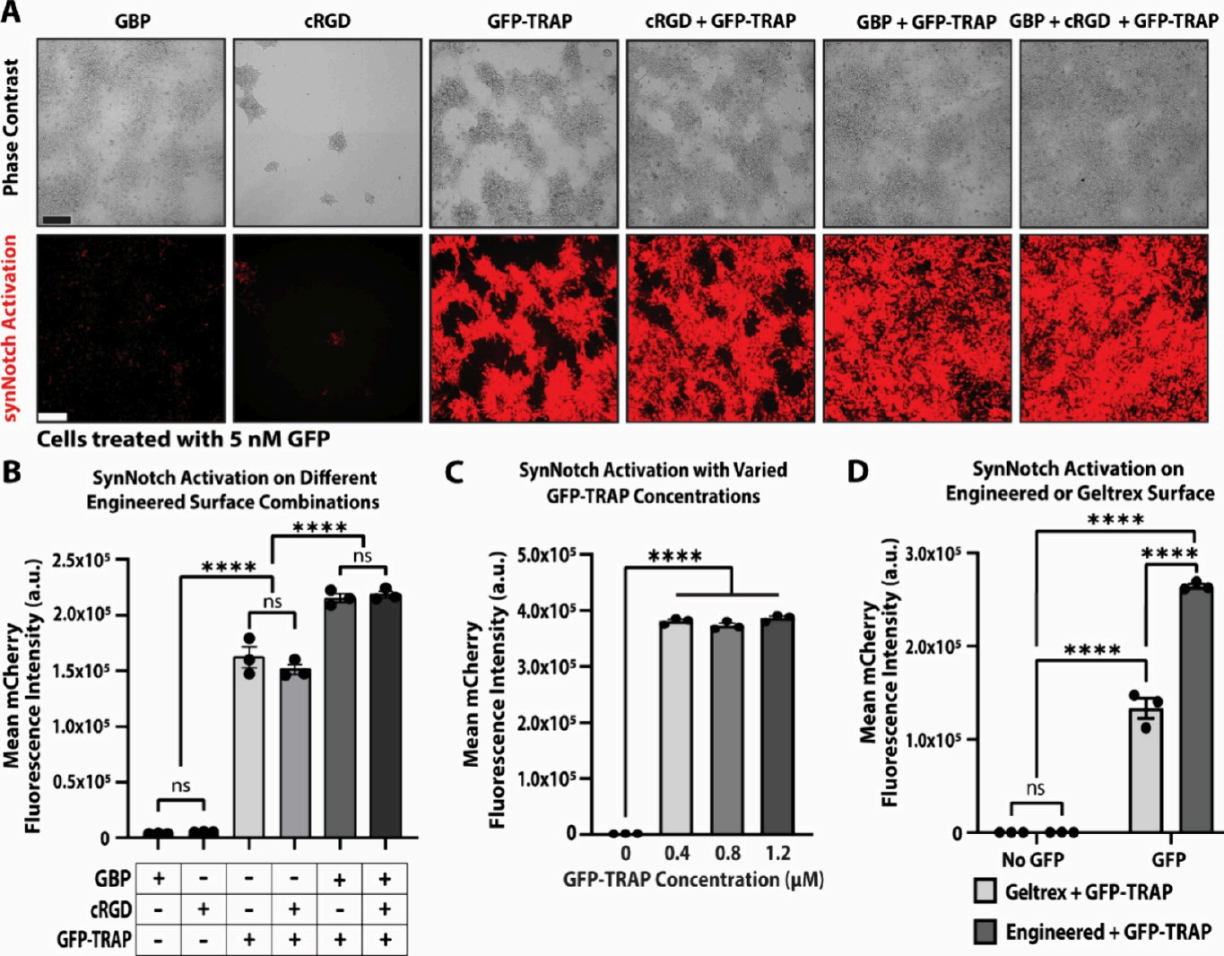
**Assessment of synNotch-PSC adhesion and activation on varied substrata.** (A) Representative phase contrast and fluorescence microscopy images of GFP-responsive reporter-synNotch H9 hESCs dosed with 5 nM GFP after being plated on various functionalized substrata for 4 days. Cells were plated at high density to simulate PSC-differentiation protocols. Single peptide substrata were composed of 5 μM GBP, 5 μM cRGD, or 0.8 μM GFP-TRAP. Dual peptide surfaces were composed of 5 μM GBP or cRGD and 0.4 μM GFP-TRAP. The tri-peptide substratum was composed of 5 μM GBP, 2.15 μM cRGD, and 0.4 μM GFP-TRAP. (B) Mean mCherry fluorescence intensity of GFP-responsive reporter-synNotch H9 hESCs dosed with 5 nM GFP on different substrata combinations after 4 days. One-way ANOVA with Tukey’s multiple comparisons post hoc. ****p < 0.0001. (C) Mean mCherry fluorescence intensity of GFP-responsive reporter-synNotch H9 hESCs on the tri-peptide surface dosed with 5 nM GFP at various GFP-TRAP concentrations on day 2 of culture. One-way ANOVA with Tukey’s multiple comparisons post hoc: **** *p* < 0.0001. (D) Mean mCherry fluorescence intensity of GFP-responsive reporter-synNotch H9 hESCs plated on the tri-peptide substratum or a Geltrex-coated surface mixed with GFP-TRAP with and without 5 nM GFP supplementation. Two-way ANOVA with Tukey’s multiple comparisons post hoc: ****p < 0.0001. In all plots, *n* = 3 replicate wells; error bars indicate SEM. Scale bars = 200 μm.

As PSCs are anchored in the stem cell niche by a variety of adhesion molecules including cadherins, integrins, and other cell-surface macromolecules,^55−59^ they require a basement membrane coating, typically Matrigel or Geltrex, for subcultivation. However, these commercially available substrata are not fully defined, nor do they provide an avenue to readily incorporate synNotch ligands or ligand-capturing motifs. To overcome this problem, we developed a fully defined, peptide-based substratum that provides PSC attachment sites as well as a synNotch ligand-capturing motif, making the platform compatible with PSC culture and differentiation, while maintaining its ability to immobilize soluble activating ligand to initiate synNotch signaling.^36^ The fully defined substratum was composed of two adhesion peptides derived from vitronectin and fibronectin—glycosaminoglycan (GAG) binding peptide (GBP)^60^ and cyclic arginine-glycine-aspartate (cRGD),^61^ respectively—in addition to GFP-TRAP, which captures the soluble, bioinert GFP that serves as a synNotch ligand (Figure 2A). GBP is a heparin-binding peptide that interacts with GAGs on the stem cell surface and has been shown to support PSC growth and propagation for up to three months in monolayer.^60^ cRGD is an adhesion motif that has a high affinity for α_V_β_3_ and α_V_β_5_ integrins on the surface of cells and has been widely exploited for cell adhesion and signaling.^62−66^ Furthermore, cRGD in combination with GBP has been shown to support stem cell differentiation with comparable levels of adhesion to Matrigel-coated surfaces.^61^

In this work, we first sought to understand the contributions of each component in the peptide-based substratum by testing different combinations of GBP, cRGD, and GFP-TRAP to assess PSC adhesion and synNotch activation with the goal of extending our platform’s utility for prolonged PSC differentiation protocols. For initial experiments, the payload transgene regulated by synNotch in H9 hESCs was the fluorescent reporter protein mCherry, which could be monitored via microscopy and flow cytometry to determine receptor activation efficiency. Reporter synNotch-hESCs were plated on a variety of substrata in mTeSR Plus medium supplemented with 10 μM ROCK inhibitor (Y-27632). Short-term, four-day experiments revealed a dependency of cell adhesion and synNotch activation levels on substratum composition (Figure 2A, Supp Figure 1A, B). Whereas cells on the GBP surface adhered for the duration of the four-day culture, cRGD-only surfaces failed to promote hESC adhesion. Unexpectedly, surfaces treated with GFP-TRAP alone facilitated cell attachment (Figure 2A), but only in conditions with 5 nM GFP supplementation (Supp. Figure 1A). Similarly, surfaces modified with GBP+GFP-TRAP rendered sustained cell adhesion in conditions with (Figure 2A) and without GFP (Supp. 1A), but adhesion to the cRGD+GFP-TRAP condition depended on the presence of GFP to mediate cell attachment (Figure 2A, Supp. 1A). The trifunctionalized surface combining GBP, cRGD, and GFP-TRAP rendered adhesion of cells for the duration of the culture, irrespective of supplementation with GFP ligand (Figure 2A, Supp. 1A). Our results are consistent with prior reports in which surfaces coated with GBP promoted adhesion of cells in the pluripotent state, whereas cRGD alone was ineffective at enabling hESC attachment.^60^ Further, these results show that GFP-TRAP does not negatively influence hESC adhesion, but rather it potentiates adhesion of synNotch-hESCs exposed to soluble synNotch ligand.

In addition to determining the influence of each peptide on substratum-mediated hESC adhesion, we evaluated levels of ligand-dependent synNotch activation on each substratum type. Fluorescence microscopy (Figure 2A) and flow cytometry analysis (Figure 2B, Supp. 1B) reveal potent expression of the synNotch-regulated mCherry on each surface incorporating GFP-TRAP, with the highest activation achieved on surfaces including both GBP and GFP-TRAP. In the absence of GFP-TRAP, GFP supplementation resulted in only weak mCherry expression, in agreement with our prior work describing the MATRIX platform.^36^ Further, in the absence of GFP, synNotch transgene expression was negligible (Supp. 1A). These results suggest that an optimized surface that enables hPSC adhesion and potent, GFP-driven synNotch signaling requires both GBP and GFP-TRAP, and that addition of cRGD does not have a deleterious impact on ligand-dependent synNotch activation.

Next, we turned our attention to optimizing the amount of GFP-TRAP incorporated in a tri-peptide-functionalized substratum consisting of GBP, cRGD, and GFP-TRAP. In prior work, we discovered that increasing the level of GFP-TRAP on cell culture surfaces led to enhanced synNotch signaling in engineered fibroblasts.^36^ Therefore, we interrogated whether varied GFP-TRAP levels influenced synNotch-PSC activation. In our initial MATRIX platform, we utilized 0.8 μM GFP-TRAP in combination with 5 μM GBP and 2.15 μM cRGD,^36^ thus we plated reporter synNotch-hESCs on substrata composed of 5 μM GBP, 2.15 μM cRGD, and either 0, 0.4, 0.8, or 1.2 μM GFP-TRAP. Cells were cultured in mTeSR Plus supplemented with 10 μM ROCK inhibitor. After 2 days of culture in 5 nM GFP, we found that all three concentrations of GFP-TRAP resulted in similar levels of synNotch activation, as indicated by mean mCherry fluorescence intensity values measured by flow cytometry (Figure 2C, Supp. 1C, D). As increasing GFP-TRAP concentration had minimal effect on synNotch activation, and reasoning that a substratum composed of lower levels of GFP-TRAP (and therefore relatively higher levels of adhesion peptide) would facilitate the most robust and durable PSC adhesion, we selected the 0.4 μM GFP-TRAP formulation, half the concentration of the initial MATRIX platform, for the engineered surface used in subsequent studies.

We then compared the performance of the optimized tri-peptide surface of GBP+cRGD+GFP-TRAP to a surface functionalized with the commercially available and widely used basement membrane Geltrex mixed with GFP-TRAP. Upon addition of GFP, we found Geltrex+GFP-TRAP surfaces gave rise to moderate synNotch activation (Figure 2D, Supp. 1E, F), but with mean mCherry levels less than half that achieved with cells cultured on the engineered, tri-peptide surface. In addition, activation levels were inconsistent across the Geltrex+GFP-TRAP surface (Supp. 1E), suggesting that the Geltrex treatment of the cell culture surface precluded efficient interactions between GFP-TRAP and synNotch cells. Thus, the tri-peptide substratum was able to promote superior synNotch activation while promoting similar levels of adhesion to a commercially available basement membrane. Based on the collection of results presented here and on prior reports indicating a synergistic effect of GBP and cRGD for sustaining pluripotency while also being compatible with multiple differentiation strategies,^60,61^ we proceeded with the trifunctionalized GBP, cRGD, GFP-TRAP engineered substratum for remaining experiments.

### The PSC-MATRIX Platform Enables Temporal Control of Artificial Signaling Events

To recreate the complex signaling mechanisms associated with morphogenesis, many differentiation protocols rely on time-sensitive activation and/or inhibition of signaling pathways through a series of small molecule treatments.^15,16,18,37,38^ These approaches to producing PSC-derived tissues highlight the importance of temporal control of morphogen modulation during PSC differentiation. Because the PSC-MATRIX platform leverages a soluble input instead of a ligand-conjugated substratum, the platform is in principle compatible with arbitrarily selected synNotch induction time-points. To verify whether PSC-MATRIX indeed accommodates various GFP-treatment regimens, we plated reporter synNotch-hESCs at a high density (150,000 cells/cm^2^, simulating densities of differentiation protocols) on the tri-peptide substratum in mTeSR plus medium with 10 μM ROCK inhibitor. The medium was then supplemented with 5 nM GFP at 0, 24, or 96 h after plating and mean mCherry pixel intensity was tracked for 10 days.

**Figure 3:**
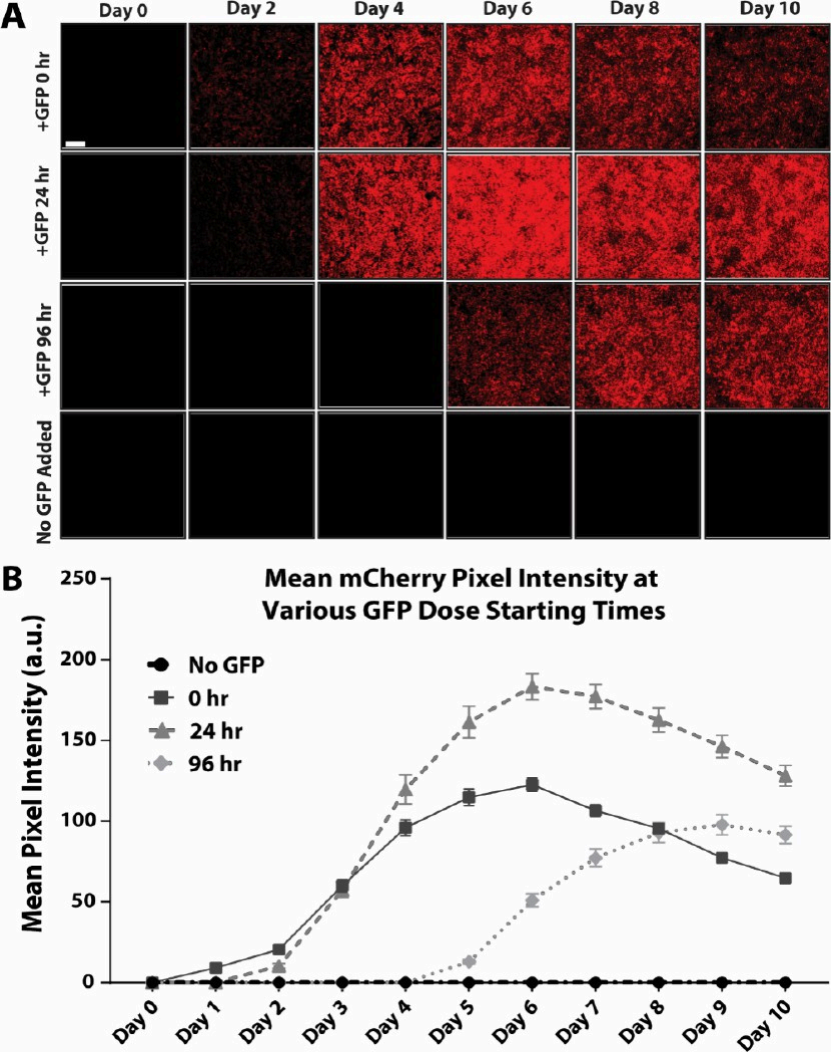
**Influence of delayed ligand provision on synNotch-PSC activation levels.** (A) Representative fluorescence microscopy images of GFP-responsive reporter-synNotch H9 hESCs plated in high density on the tri-peptide substratum and dosed with 5 nM GFP at 0, 24, and 96 h after plating at days 2, 4, 6, 8, and 10. (B) Quantification of mean pixel intensity of mCherry expression from reporter-synNotch H9 hESCs without GFP or supplemented with GFP at 0, 24, or 96 h after plating over 10 days of culture in mTeSR Plus medium supplemented with 10 μM ROCK inhibitor. Medium was changed daily and GFP was added each day after the initial dose. *n* = 15, 5 fields of view from 3 replicate wells; error bars indicate SEM. Scale bar = 200 μm.

Fluorescence microscopy (Figure 3A) indicates potent ligand-dependent expression of synNotch-regulated mCherry following GFP supplementation, independent of the time of GFP treatment. Additionally, synNotch activation was sustained for extended culture, up to 10 days, following addition of GFP in the medium. Furthermore, the cells remained adhered to the surface for the duration of the 10-day experiment regardless of GFP supplementation (Figure 3A, Supp. 2). Quantification of the mean mCherry pixel intensity (Figure 3B) surprisingly revealed enhanced maximal activation levels when GFP was dosed 24 h after plating cells as compared to GFP treatment at 0 or 96 h (Day 10: 0 h vs 24 h ****p < 0.0001, 0 h vs 96 h **p = 0.001, 24 h vs 96 h ***p < 0.0008). Tracking longitudinal mCherry intensity profiles revealed that synNotch transgene expression peaked 6 days after GFP was dosed at 0 h, and at 5 days after GFP was dosed at 24 or 96 h (Day 6: No GFP vs 0 h, ****p < 0.0001. No GFP vs 24 h, ****p < 0.0001; Day 9: No GFP vs 96 h, ****p < 0.0001). These results illustrate that PSC-MATRIX can flexibly accommodate diverse experiment initiation schemes, which may require delayed transgene induction, without compromising considerable dynamic range and maximal transgene expression levels. Despite revealing that a 24 h delay in synNotch-PSC activation renders the most robust levels of synNotch-driven transgene production, our subsequent experiments deployed a 0 h initiation time to enable rapid transgene expression and to avoid the risk of spontaneous differentiation at high cell plating densities.

### The PSC-MATRIX Platform Facilitates Artificial Signaling in both Maintenance and Differentiation Conditions

After verifying that the defined PSC-MATRIX substratum enabled efficient adhesion and privileged communication via the synNotch signaling channel, we sought to ensure the artificial surface was compatible with both hESCs and iPSCs in maintenance and differentiation media. To this end, we engineered CC3 iPSCs^43^ to express the same GFP-responsive synNotch receptor and fluorescent mCherry transgene payload as the H9 hESCs from earlier experiments. We then plated each cell line on the tri-peptide substratum in either mTeSR Plus or Essential 8 (E8) stem cell maintenance medium and N2B27 differentiation medium supplemented with 5 nM GFP. Cells were cultured for 4 days before performing flow cytometry to quantify synNotch activation via mCherry expression. Following prior reports that sustained hPSC adhesion and maintenance requires continued ROCK inhibition,^60,61^ we also tested each medium formulation with three different concentrations of Y-27632, at 10, 5, and 2.5 μM.

**Figure 4:**
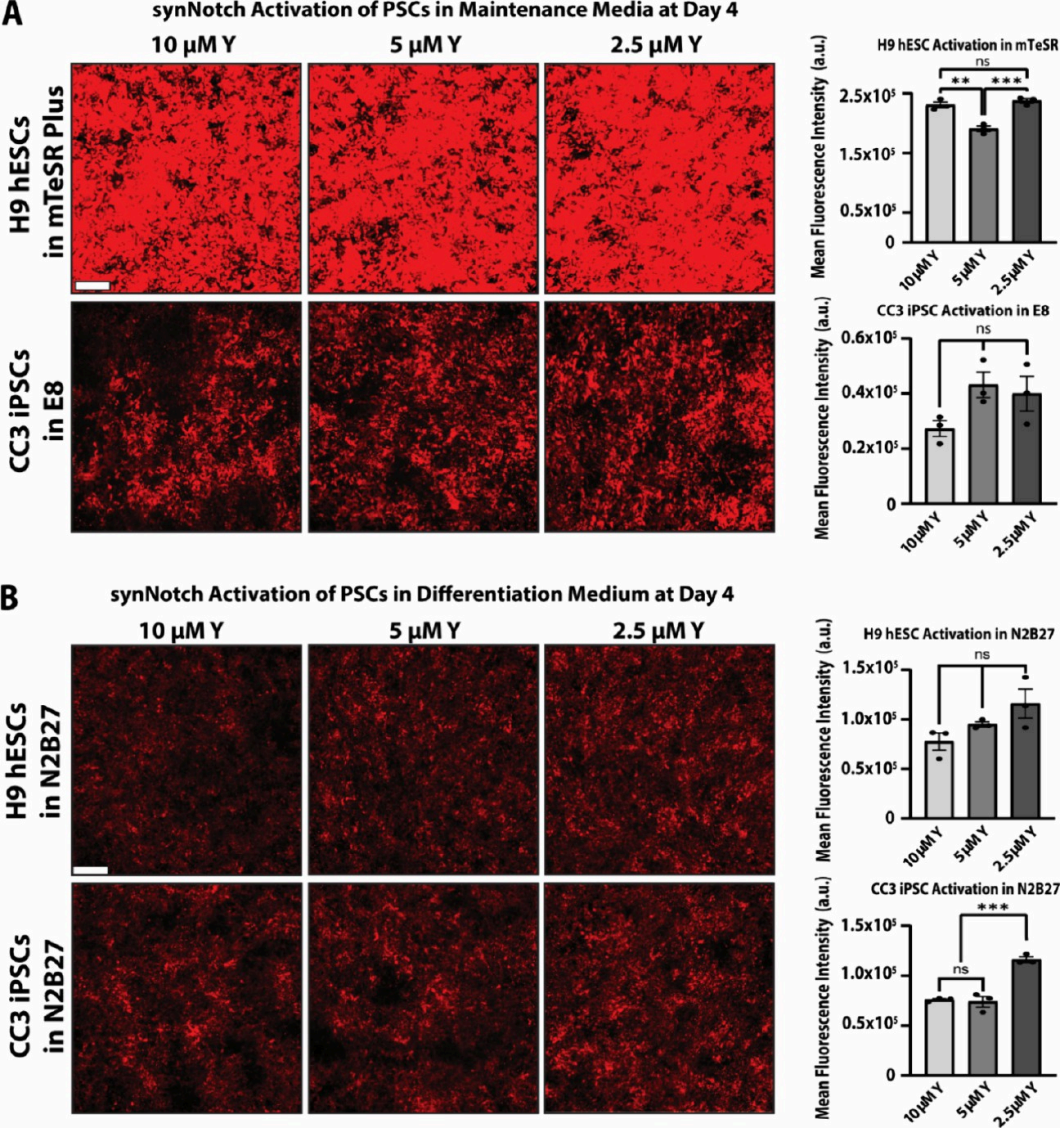
**Assessment of the ability of PSC-MATRIX to guide ligand-dependent hESC and hiPSC gene expression in both maintenance and differentiation media.** Representative fluorescence microscopy images and mean mCherry fluorescence intensity of GFP-responsive reporter-synNotch H9 hESCs and CC3 iPSCs plated in high density on the engineered substratum and maintained in (A) stem cell maintenance medium - mTeSR Plus for H9 hESCs and Essential 8 (E8) for CC3 iPSCs - or (B) N2B27 differentiation medium supplemented with 10 μM, 5 μM, or 2.5 μM ROCK inhibitor (Y-27632) for 4 days. One-way ANOVA with Tukey’s multiple comparisons post hoc: **p < 0.01, ***p < 0.001. In all plots, *n* = 3 replicate wells; error bars indicate SEM. Scale bars = 200 μm.

Both synNotch-hESCs and synNotch-iPSCs adhered to and exhibited synNotch activation on the optimized tri-peptide substratum in their respective maintenance media - mTeSR Plus for synNotch-H9 hESCs and E8 for synNotch-CC3 iPSCs - and N2B27 differentiation medium in all three ROCK inhibitor concentrations (Figure 4, Supp. 3). In mTeSR Plus, we found that synNotch-H9 hESCs displayed robust adhesion and synNotch activation. Furthermore, decreasing ROCK inhibitor concentration did not impact H9 hESC adhesion on the defined substratum and had a subtle, but statistically significant impact on synNotch activation. (Figure 4A, Supp. 3A). In E8 medium, the synNotch-CC3 iPSCs displayed robust adhesion and synNotch activation in all three ROCK inhibitor conditions (Figure 4A, Supp. 3A). Additionally, decreasing the ROCK inhibitor concentration did not result in a statistically significant difference in synNotch activation (Figure 4A).

Intriguingly, in N2B27 medium, we observed very little difference in adhesion and synNotch activation between the synNotch-CC3 iPSCs and synNotch-H9 hESCs (Figure 4B, Supp. 3B). While decreasing the concentration of ROCK inhibitor did not visibly affect iPSC or hESC adhesion to the substratum, it did correspond with a subtle increase in synNotch activation as indicated by flow cytometry (Figure 4B, Supp. 3B). Namely, synNotch-CC3 iPSCs had a subtle but statistically significant increase in synNotch activation at the lowest ROCK inhibitor concentration. Furthermore, in both maintenance and differentiation media alone, neither the H9 hESCs nor CC3 iPSCs displayed ligand-independent activation (Supp. 3). While the microscopy suggests overall weaker induction in N2B27 for both cell types, flow cytometry histograms reveal an mCherry MFI fold-change of approximately 200-fold (H9 hESCs) and 125-fold (CC3 hiPSCs) in the 0 nM versus 5 nM conditions, reflecting a dramatic and wide dynamic range of synNotch activation in N2B27 (Supp. 3A-B). Similarly, subsequent experiments with a synNotch-KOLF2.1J hiPSC line validated that PSC-MATRIX affords potent GFP-dependent artificial signaling that, while dramatic in all conditions, varies in magnitude based on medium formulation (Supp. 3C). Overall, we determined that PSC-MATRIX enables stem cell adhesion and robust activation of both synNotch-hESCs and synNotch-hiPSCs in their respective maintenance and in differentiation media.

### PSC-MATRIX Enables the Activation of Engineered PSCs in a Spatially Constrained Manner

**Figure 5:**
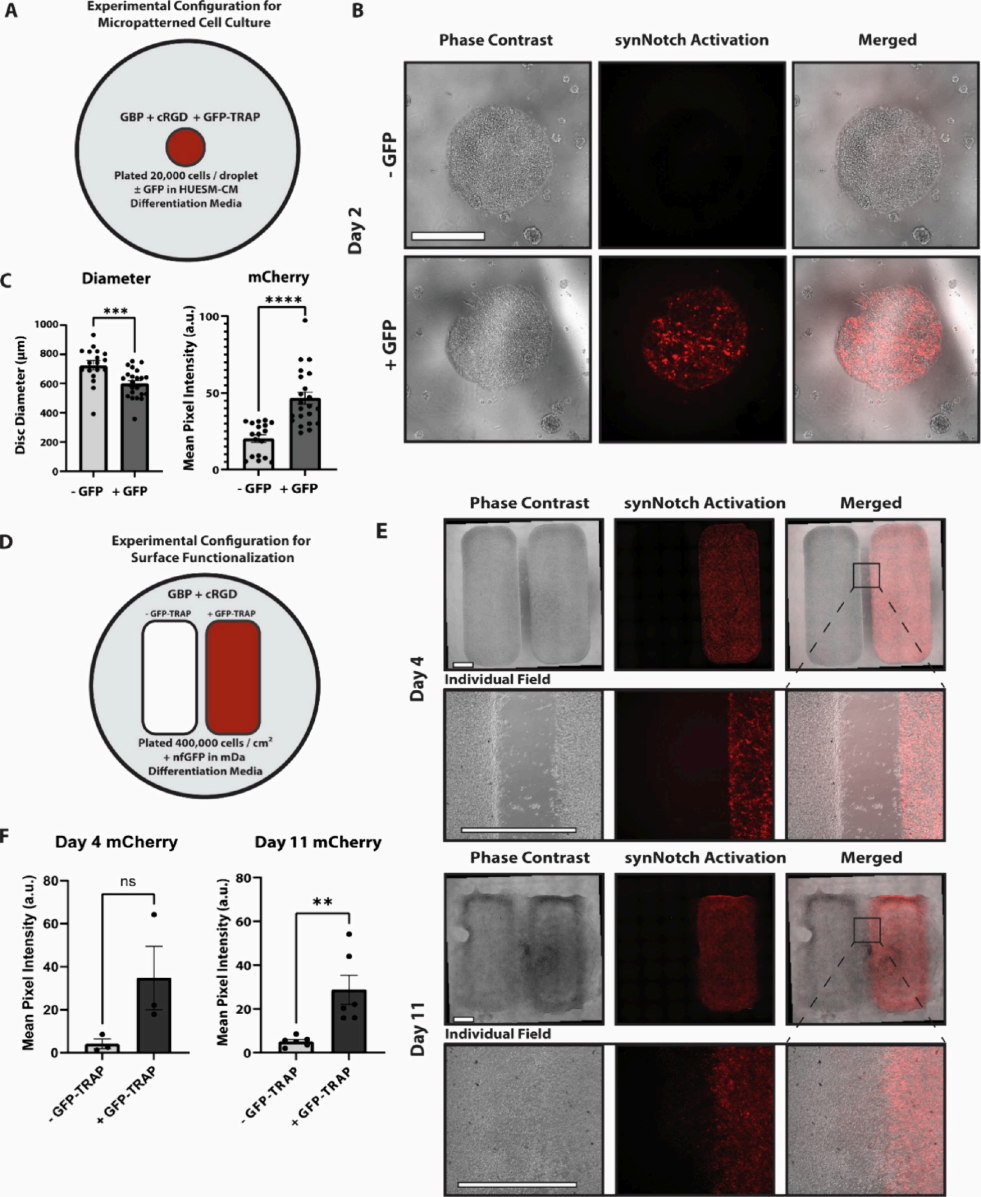
**Demonstration of the capacity of PSC-MATRIX to facilitate both stem cell micropatterning and spatially constrained artificial signaling.** (A) Experimental configuration of micropatterned cell culture with the functionalized surface in HUESM-CM differentiation medium with or without GFP. (B) Representative day 2 phase contrast and fluorescence microscopy of inducible WNT3a synNotch-hESCs depicts ligand-dependent synNotch activation via mCherry expression. Scale bars = 500 μm. (C) Quantification of average disc diameter calculated using Leica LAS X software and mean pixel intensity of mCherry fluorescence of each disc calculated using ImageJ software (+GFP *n* = 22; -GFP *n* = 18). Unpaired *t* test for statistical significance: ***p < 0.001, ****p < 0.0001; error bars indicate SEM. (D) Experimental configuration illustrating the functionalization of a 2-panel insert; the left panel was only functionalized with 5 μM GBP and 2.15 μM cRGD whereas the right panel was functionalized with the adhesion peptides in addition to 0.4 μM GFP-TRAP. mDa differentiation medium supplemented with 5 nM nfGFP was then added to the well plate. (E) Representative day 4 and day 11 phase contrast and fluorescence microscopy of inducible WNT3a synNotch-hESCs depicts ligand-dependent synNotch activation via mCherry expression only on the panel functionalized with the adhesion peptides and GFP-TRAP. Individual field images were stitched together using LAS X software. Scale bars = 1000 μm. (F) Quantification of mean pixel intensity of mCherry on the adhesion only and adhesion + GFP-TRAP functionalized panels (day 4: *n* = 3, 1 well from 3 separate experiments; day 11: *n* = 6, 3 replicate wells from 2 separate experiments). Unpaired *t* test for statistical significance: **p < 0.01; error bars indicate SEM.

Several synthetic morphogenesis studies leverage micropatterning to develop 100–1000 μm PSC discs to investigate early developmental events such as gastrulation.^15,37,39,50,67,68^ These studies reveal a radial dependence of PSC differentiation after bulk application of morphogens to these cultures, where cells within the center of discs demonstrate features of ectoderm while cells in the adjacent and peripheral regions exhibit markers of mesendoderm and definitive endoderm, respectively.^15,37,50^ In contradistinction, recent studies have illustrated that cell fate specification within these discs differ when cells produce differentiation-stimulating morphogens themselves rather than receiving these defining signaling factors as medium supplements,^69^ but the mechanisms at play remain ill-defined. Thus, we sought to determine whether we could extend PSC-MATRIX to serve as a tool to conduct such experiments. We interrogated whether the platform could be used to produce discs in which artificial signaling mediates orthogonal stimulation of morphogen production. To do this, we patterned the tri-peptide substratum to constrain cell adhesion and synNotch activation in defined areas within a well (Figure 5A). Our process resulted in synNotch-hESC discs with diameters ranging from 300 to 900 μm (Figure 5C), similar to widely used synthetic morphogenesis protocols.^15,37,39,50,67,68^ To show feasibility of using these cells in early peri-gastrulation studies, we engineered and used synNotch-hESCs that express WNT3a along with mCherry in response to GFP activation. Cells were cultured in the gastrulation differentiation medium HUESM-CM^37^ (plus 10 μM ROCK inhibitor) with or without GFP. After 48 h in culture, the micropatterned WNT3a-mCherry synNotch-hESCs remained adherent in confluent micropatterned discs, with only GFP-treated groups displaying synNotch-driven mCherry expression (Figure 5B, C). Furthermore, activation of synNotch cells engineered to express the morphogen WNT did not have an overtly negative influence on adherence of the micropatterned colonies over 48 h in differentiation medium. These results indicate that PSC-MATRIX can be deployed to drive cell-secreted morphogen expression in the context of micropatterns mimicking gastrulation. Thus, PSC-MATRIX may serve as a tool to conduct experiments investigating the role of morphogen induction from within such micropatterned colonies.

In our prior work, we utilized the juxtacrine-like responsiveness of synNotch to spatially localize receptor activation to bulk soluble cues in engineered fibroblasts.^36^ As developmental events rely on secreted morphogens that are often localized to a single region, called signaling centers,^70,71^ we set out to determine whether we can establish PSC-derived artificial signaling centers using the privileged cell-material communication channel of PSC-MATRIX. Thus, we used an Ibidi culture insert to functionalize distinct regions of a culture well with the dual adhesion peptides GBP and cRGD, and we decorated a distinct, adjacent region with the tri-peptide surface that includes GFP-TRAP (Figure 5D). Cells were then plated in both chambers in an N2B27-based differentiation medium, and subsequently the Ibidi insert was removed. A uniform bath of 5 nM of the Y67F variant of GFP was added to activate synNotch signaling. This GFP mutant, which we call nonfluorescent GFP (nfGFP), preserves the epitopes recognized by GFP-TRAP and the LaG16-synNotch but abolishes the fluorescence of the protein.^47^ Results illustrate that the WNT3a-mCherry synNotch-hESCs remained adherent to both functionalized regions, with only the domain decorated with GFP-TRAP displaying synNotch-driven mCherry expression (Figure 5E, F). Transgene mCherry expression was sustained for the 11-day duration of the experiment in differentiation medium (Figure 5E, F). By day 11, cells were able to traverse the gap separating the dual-adhesion peptide-decorated surfaces; despite this, mCherry-positive cells were not apparent in the region activated with only adhesion peptides and lacking GFP-TRAP (Figure 5E). These results illustrate that the selective functionalization of PSC-MATRIX enables spatial control of orthogonal, synNotch-driven transgene expression, opening the door to use this platform to mimic morphogen-secreting signaling centers to direct stem cell differentiation.

### PSC-MATRIX Platform Potentiates PSC Morphogenesis via Artificial Signaling

After verifying that the fully synthetic, tri-peptide substratum enabled robust adhesion and synNotch activation in reporter synNotch-ESCs and synNotch-iPSCs, we sought to use the capabilities of orthogonal signaling offered by this platform to initiate morphogenetic programs in synNotch-PSCs. One of the earliest steps in stem cell fate determination occurs during gastrulation when the epiblast organizes into the three germ layers–the ectoderm, mesoderm, and endoderm. This process is orchestrated by the morphogens WNT, BMP, and NODAL.^12,67,72,73^ Early markers of gastrulation include Brachyury (BRA, a primitive streak and mesendodermal marker), vascular endothelial growth factor receptor 2 (VEGFR2, a mesodermal marker), SOX2 (a pluripotency marker that maintains expression during ectoderm differentiation), and SOX17 (an endodermal marker).^37,50−52^ To assess the ability of PSC-MATRIX to guide and support short-term differentiation, WNT3a-mCherry synNotch-hESCs were put through a 48 h peri-gastrulation differentiation. Prior to initiating morphogenetic events, the WNT3a-mCherry synNotch-hESCs displayed positive expression of the conventional pluripotency markers stage-specific embryonic antigen-4 (SSEA 4) and TRA-1–81 (Podocalyxin) (Supp. 4A).^74^ WNT3a-mCherry synNotch-hESCs were plated on the tri-peptide substratum in HUESM-CM differentiation medium^37^ supplemented with 10 μM ROCK inhibitor, and a subset of the wells were treated with 5 nM nfGFP or 5 μM CHIR99021 (CHIR), a WNT activator. For comparison, WNT3a-mCherry synNotch-hESCs were plated on the tri-peptide substratum and maintained in mTeSR Plus maintenance medium supplemented with 10 μM ROCK inhibitor without nfGFP. synNotch-hESCs were cultured for 48 h and the medium was replaced after 24 h. After 48 h of culture, all groups were fixed and immunolabeled for the differentiation markers BRA, VEGFR2, SOX2, and SOX17.

**Figure 6:**
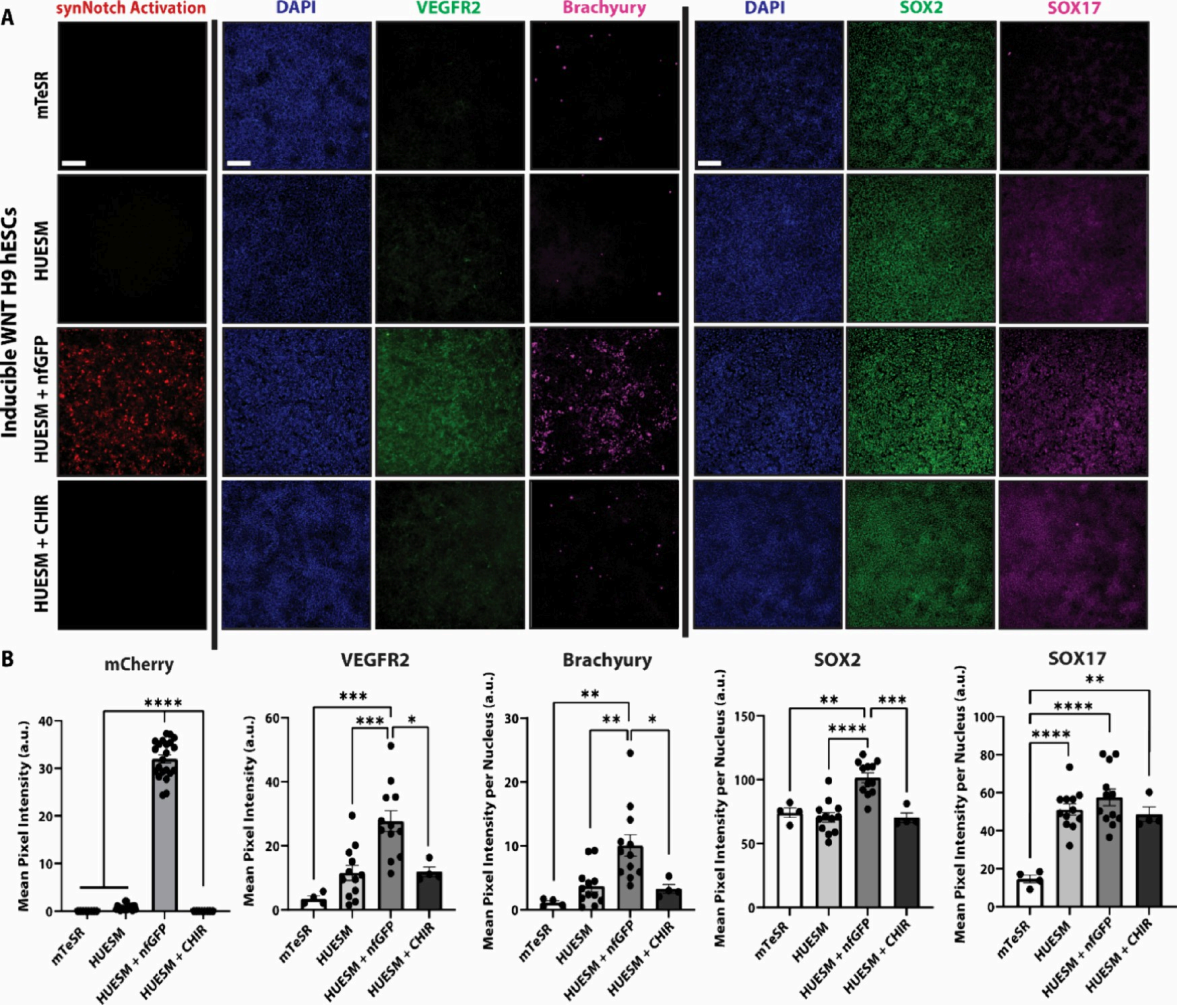
**Mesendodermal differentiation of stem cells enabled by PSC-MATRIX.** (A) Representative fluorescence microscopy of GFP-responsive WNT3a synNotch-hESCs that underwent a 48 h gastrulation differentiation protocol on the tri-peptide substratum. The cells were maintained in HUESM-CM medium with 10 μM ROCK and were left untreated, treated with 5 nM nfGFP, or treated with 5 μM CHIR. For comparison, WNT3a synNotch-hESCs were maintained on the tri-peptide substratum in mTeSR PLUS maintenance medium with 10 μM ROCK (*n* = 4). The first panel of images depicts ligand-dependent synNotch activation via fluorescence microscopy, as mCherry is coexpressed with WNT3a. The remaining panels correspond with the immunofluorescence labeling for BRA, VEGFR2, SOX2, and SOX17–markers for germ layer differentiation. BRA and VEGFR2 immunolabeling were performed in the same wells, and SOX2 and SOX17 immunolabeling were performed in a separate set of wells. (B) Quantification of mean pixel intensity of mCherry (*n* = 22), BRA, VEGFR2, SOX2, and SOX17 (± nfGFP *n* = 12, CHIR and mTeSR *n* = 4) using ImageJ. One-way ANOVA with Tukey’s multiple comparisons post hoc: *p < 0.05, **p < 0.01, ***p < 0.001, ****p < 0.0001; error bars indicate SEM. Scale bars = 200 μm.

After 48 h of culture, the WNT3a-mCherry synNotch-hESCs remained adherent in all conditions, with only nfGFP-treated groups displaying synNotch-driven mCherry expression (Figure 6, Supp. 4B). Furthermore, GFP-induced, synNotch-driven morphogen expression was sufficient to encourage the WNT3a-mCherry synNotch-hESCs to differentiate into a BRA- and VEGFR2-positive population with increased levels of SOX2 and SOX17 expression, especially when compared to the mTeSR group, which is indicative of a transition to an early peri-gastrulation cell state (Figure 6).^37,50−52^ In comparison, the WNT3a-mCherry synNotch-hESCs cultured in HUESM-CM differentiation medium without nfGFP did not express BRA, and VEGFR2 expression was markedly lower in the absence of activating ligand. While the untreated cells in HUESM-CM still expressed SOX2 and SOX17, the mean pixel intensity did trend lower than the nfGFP treated group. The elevated levels of the endodermal marker SOX17 may be due, in part, to the supplementation of FGF in the HUESM-CM differentiation medium, as it is a key signaling molecule known to give rise to definitive endoderm.^75^ Interestingly, the CHIR-treated group displayed minimal BRA and VEGFR2 expression. The difference in marker expression between the nfGFP-treated and CHIR-treated groups could be explained by the timing of our assessment, as BRA expression is biphasic, occurring during primitive streak formation followed by a decline prior to a boost in expression again during notochord development.^51,76^ A differential level of potency between ectopic WNT production and CHIR-mediated WNT-signaling may result in differential peaks of BRA expression. Altogether, these results show that the optimized PSC-MATRIX platform can activate orthogonal artificial signaling circuits in synNotch-PSCs to inducibly drive stem cells to transition toward early mesendodermal fates, reminiscent of 2D, in vitro models of peri-gastrulation.^15,37,50,51,68^

**Figure 7:**
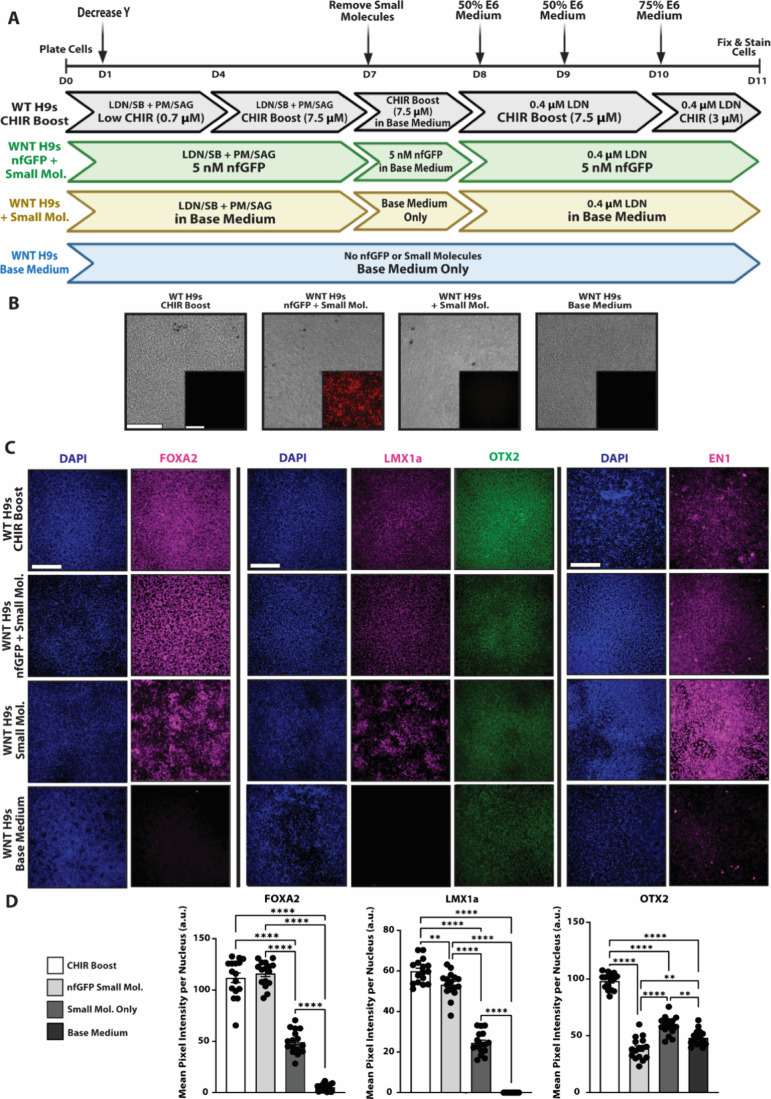
**Assessment of midbrain dopaminergic progenitor markers in WNT3a-synNotch-PSCs**. (A) Timeline for mDa differentiation and the various tested culture conditions. Inducible WNT3a synNotch-hESCs underwent an 11-day mDa differentiation on the engineered substratum and were compared to WT H9s cultured on Geltrex undergoing the same differentiation guided by CHIR supplementation. (B) Phase contrast and fluorescence microscopy depicts ligand-dependent synNotch activation via mCherry expression which is coexpressed with WNT3a. (C) Representative immunofluorescence for FOXA2, LMX1a, OTX2, and EN1 expression on day 11. LMX1a and OTX2 immunolabeling were performed in the same wells, whereas FOXA2 and EN1 immunolabeling were each performed in separate sets of wells. (D) Quantification of mean pixel intensity of FOXA2, LMX1a, and OTX2 using ImageJ (*n* = 16). One-way ANOVA with Tukey’s multiple comparisons post hoc: **p < 0.01, ***p < 0.001, ****p < 0.0001; error bars indicate SEM. Scale bars = 200 μm.

To further assess the ability of the optimized tri-peptide substratum to guide ligand-inducible differentiation programs that require longer culture durations, we put the inducible WNT3a synNotch-hESCs through an 11-day midbrain dopaminergic neuron (mDa) progenitor differentiation protocol.^38^ Signaling via WNT1, WNT3a, and WNT5a plays an important role in midbrain development, as WNT patterns the anteroposterior axis in the forebrain and midbrain, establishes the midbrain-hindbrain boundary organizer, and promotes mDa neuron specification and neurogenesis.^77−79^ WNT3a synNotch-hESCs were thus cultured on the substratum and maintained in medium supplemented with combinations of the dual SMAD inhibitors LDN193189 dihydrochloride (LDN) and SB431542 (SB), and Sonic Hedgehog agonists purmorphamine (PM) and Smoothened agonist (SAG), with or without 5 nM GFP to induce WNT3a production. For comparison, WT H9s were subjected to a modified, biphasic WNT protocol^38^ exploiting the aforementioned small molecules along with CHIR99021 (CHIR) as a WNT activator on a Geltrex-treated surface. For control conditions, WNT3a synNotch-hESCs plated on the substratum were treated with SB, LDN, PM, and SAG alone (i.e., no WNT activation) or they were plated on the substratum without any small molecules or nfGFP (i.e., base medium only) (Figure 7A). After 11 days, cell differentiation was characterized by immunolabeling for canonical mDa neuron progenitor markers FOXA2, LMX1a, OTX2, and EN1, previously used to validate PSC-derived mDA progenitors.^38,53,54^

Results show potent, ligand-dependent synNotch activation through the end of the differentiation protocol (Figure 7B, Supp. 5A), indicating sustained expression of the WNT3a transgene cassette. Throughout the 11-day differentiation protocol, cells remained adhered to the tri-peptide substratum at a level comparable to those cultured on Geltrex, as indicated by uniform DAPI staining as well as phase contrast images (Figure 7C, Supp. 5). GFP-induced WNT3a expression in combination with small molecules SB, LDN, PM and SAG gave rise to FOXA2+, LMX1a+, and EN1+ populations of cells that were comparable to the CHIR-supplemented WT H9s (Figure 7C, D, Supp. 5B). In contrast, cells cultured in the SB, LDN, PM, and SAG cocktail without a WNT activator (i.e., CHIR or nfGFP) were deficient in FOXA2 expression and adopted a distinct EN1 distribution as displayed by immunofluorescence. Cells cultured in the absence of both WNT activator and small molecules were deficient in all markers of mDA progenitor differentiation other than OTX2. Surprisingly, the level of OTX2 expression in the ligand-treated PSC-MATRIX cells was modest and comparable to control conditions without WNT activator. Combined with the high levels of FOXA2, LMX1a, and EN1, this suggests cells may have been transiting toward the OTX2-low A9 dopaminergic neuron subtype.^80^ Altogether, these results indicate that the PSC-MATRIX platform incorporating the WNT3a synNotch-H9 hESCs is able to support and coordinate artificially induced morphogenesis to render mDa neuron progenitors, recapitulating key features of a recently established protocol involving CHIR supplementation.^38^

## Discussion

Here, we present a PSC-specific version of the MATRIX platform, PSC-MATRIX, that supports prolonged hESC and iPSC culture and extends its utility for regulating multiple morphogenetic programs via material-mediated artificial signaling in synNotch-engineered hPSCs. We show that the composition of the peptide surface can dramatically influence PSC adhesion and synNotch activation with the tri-peptide substratum, composed of GBP, cRGD, and GFP-TRAP, displaying durable cell adhesion and potent, ligand-dependent synNotch activation. While synNotch activation was possible when GFP-TRAP was combined with Geltrex (a commercial basement membrane extract from Engelbreth-Holm-Swarm tumors), we measured dramatically reduced and nonuniform synNotch activation as compared to the tri-peptide surface. Thus, the defined tri-peptide substratum is expected to offer benefits over similar basement membrane extracts commonly used with hPSCs, including Geltrex or Matrigel, which are composed of similar ECM proteins. Results also highlight that the platform enables flexible activation windows, in which experimentalists can induce synNotch signaling via GFP-supplementation at time points up to 4 days after plating PSCs. Furthermore, we show that the artificial surface is compatible with both hESC and iPSC culture in maintenance and differentiation media, while our results indicate that levels of activation are influenced by medium composition and cell type. We also show the capacity of the platform to spatially constrain artificial signaling events by capitalizing on our privileged cell-material communication channel. Finally, we show the capability of orthogonal signaling mediated by the PSC-MATRIX platform to initiate multiple differentiation strategies in synNotch-PSCs, including establishment of peri-gastrulation fates as well as mDa neuron progenitor differentiation. Thus, we show the utility of PSC-MATRIX as a platform to probe developmental events in a material-dependent manner to activate morphogenetic programs in defined locations in response to orthogonal signaling channels.

Our prior work foreshadowed the utility of MATRIX for guiding PSC differentiation by driving transcription factor-mediated stem cell specialization. An important advance of the current work is the extension of artificial cell signaling via the juxtacrine-like synNotch channel to drive PSC differentiation by morphogen expression rather than directed differentiation. During embryonic development, morphogen signaling to PSCs occurs through complex mechanisms, including those that fit RD, PI, and lateral inhibition models to organize PSCs to migrate and establish hierarchical tissues.^12−14^ Recent studies investigating PSC patterning in RD systems have given insight into how PSCs interpret positional information within morphogen gradients based on both concentration and duration of morphogen exposure.^15,81−84^ While these studies have been informative, they exploit bulk supplementation of recombinant factors or chemical surrogates in stem cell culture media, which bypasses key initiating events from cell signaling centers that are intrinsic to development.^70,71^ Building on these studies, several synthetic biology approaches have been implemented to more faithfully recapitulate morphogenetic programs brought about by signaling centers to dynamically regulate transgene expression and implement feedback-controlled signaling cascades.^25,28,33−35,85,86^ However, these synthetic approaches did not leverage PSCs to investigate how these artificial gene circuits influenced stem cell patterning and lineage commitment. Therefore, by refining the MATRIX platform for improved performance with PSCs, we show an ability to integrate PSC culture with artificial signaling circuits to successfully regulate early peri-gastrulation and mDA neuron progenitor differentiation.

Several seminal studies have leveraged bioactive surfaces to investigate the signaling architectures that underly early developmental processes.^15,37,39,50,68,69,87^ These studies have enabled detailed dissection of the responses of cell collectives to globally provided, soluble morphogen inputs, including the propagation of BMP and/or WNT signaling through 80–1000 μm diameter PSC colonies which leads to the establishment of reproducible, radially patterned ectoderm, mesoderm, and endoderm cell populations.^15,37,39,50,67^ Typically, the bioactive surfaces used in these studies dictate cell adhesion and enable reliable colony patterning. One recent implementation of this strategy showed that the resultant radially patterned germ layer formation differs dramatically when BMP4 is cell-secreted versus supplied as a medium supplement.^69^ These results highlight the need for synthetic reconstitution studies that enable orthogonally activated, spatially constrained expression of morphogens from the PSC population. Here, we demonstrate the power to spatially localize PSC morphogen transgene expression by adding a functional peptide to the bioactive surface that enables the capture and presentation of an orthogonal synNotch ligand to cells capable of artificially generating morphogen expression. We show that this privileged communication channel can be utilized to spatially constrain synNotch-PSCs in micropatterned discs and limit synNotch activation by differentially functionalizing regions of a well plate. Furthermore, synNotch-driven morphogen expression was competent to drive numerous cell fates as a consequence of expression of WNT3a activation in monolayer. In future studies, we aim to apply these patterning techniques to further enumerate the morphogenetic outcomes (i.e., fate transitions and cell migration) of spatially arranged organizing centers that leverage signaling architectures defined by cell-secreted morphogen production. Furthermore, while this work was limited in that we only evaluated differentiation of synNotch-H9 hESCs, future studies will assess the ability of PSC-MATRIX to guide differentiation of other widely used hPSC cell lines, including the KOLF-2.1J line^45^ that has recently gained widespread adoption in the neurodegeneration community, as well as other lines derived from donors of different ages and from various tissues of origin. Finally, the outcomes of PSC-MATRIX differentiation paradigms may be easily altered by exchanging synNotch transgenes to other morphogens critical for target cell fates by substituting, for example, BMP4 for WNT3a in peri-gastrulation studies or WNT1 for WNT3a for mDA progenitor specification. Such studies will enable interrogation of the impact of patterned, locally constrained expression profiles of various genes on fate acquisition.

While PSC-MATRIX incorporates an artificial receptor to control cellular behaviors in a material-mediated manner, other nonreceptor synthetic biology platforms have shown potential for regulating PSC morphogenesis. For instance, microRNA (miRNA) switches have been used in PSCs to detect cell states and to enrich differentiated cells within a heterogeneous population.^88^ Similar miRNA-based circuits have also been used to regulate protein secretion to control the composition of differentiating PSCs.^69^ Since this approach is mediated by endogenous miRNA levels, it does not rely on exogenous cues, such as the GFP ligand used in this work; however, these circuits do require extensive screening and testing to obtain a library of relevant miRNAs to render desired cell differentiation outcomes. Furthermore, optogenetic approaches have been used to recreate morphogenetic patterning programs for embryonic development^20^ and to develop an artificial signaling center for dorsal-ventral forebrain patterning *in vitro*.^89^ While engineering PSCs to be light-responsive enables precise spatiotemporal control of signaling programs, it requires customized equipment to produce and constrain specific wavelengths of light to activate differentiation programs. In comparison, the PSC-MATRIX platform does not require any special equipment, yet it enables temporal control of artificial signaling events via the ligand capturing motif and delayed addition of activating ligand. Additionally, as demonstrated here, it can also be combined with micropatterned cell culture or commercially available well inserts to fine-tune spatial control of a synthetic signaling circuit.

## Conclusions

In conclusion, we present a PSC-specific version of our MATRIX platform, PSC-MATRIX. This platform is composed of synNotch cells cultured on a defined substratum that prolongs cell adhesion while enabling robust activation of synNotch-engineered PSCs to regulate morphogenetic programs. This platform facilitates a build-to-understand approach to morphogenesis by implementing synthetic biology tools to build artificial signaling centers. Such centers, enabled through cell-material coengineering, can be used to drive potent cell differentiation and to unravel nuances in the molecular mechanisms behind PSC fate specification. We show that our defined, tri-peptide substratum is compatible with both hESCs and iPSCs in their preferred maintenance and differentiation media, demonstrating its broad applications for PSC culture and differentiation. Furthermore, our substratum contains two adhesion peptides that could easily be swapped out or combined with other adhesion peptides to repurpose the defined substratum for application-specific uses. For instance, N-cadherin, a transmembrane adhesion protein that mediates cell–cell adhesion, replaces E-cadherins on the surface of PSCs during neurulation and helps support neural progenitors.^90,91^ As peptides derived from N-cadherin have previously been used to support PSCs during neuronal differentiation,^92,93^ it is possible that incorporating N-cadherin peptides into our substratum could support PSCs while investigating long-term neuronal morphogenetic programs. Thus, such surface modifications may augment the capability of PSC-MATRIX to support specialized stem cell applications. The third peptide in our substratum was GFP-TRAP which anchored the activating ligand, GFP, to induce our synNotch signaling circuits in a temporally controlled manner. We envision future iterations of PSC-MATRIX to incorporate capturing motifs for additional synNotch ligands. One possible ligand would be mCherry, which we and others have shown serves as an orthogonal ligand to GFP that can independently regulate the response of a second artificial receptor in engineered cells.^25,36^ This would enable manipulation of multiple morphogens simultaneously. Further, other developmentally relevant pathways, including the Notch-targeted HES and HEY transcriptional programs, could be induced via orthogonal inputs compatible with PSC-MATRIX. Finally, we demonstrated the power of the PSC-MATRIX platform to regulate morphogenetic programs in synNotch-engineered PSCs in a material-mediated manner. Here, we showed the PSC-MATRIX platform could initiate morphogenetic programs with WNT3a to stimulate PSC differentiation to mesendodermal fates, reminiscent of differentiation that occurs during peri-gastrulation. Additionally, it was able to promote an 11-day mDa neuron progenitor differentiation where GFP-induced WNT3a expression—in combination with dual SMAD inhibitors and SHH agonists—drove mDa neuron differentiation to a comparable degree as WT hPSCs in the same conditions supplemented with the WNT activator CHIR. Overall, our optimized MATRIX platform, PSC-MATRIX, offers a facile synthetic approach to morphogenesis that can be adapted for multiple lineages for a rational design, build, and analyze method to interrogate how cell-secreted morphogen signaling governs PSC differentiation.

## Supporting Information Available

The following files are available free of charge. Additional experimental results, including supporting microscopy images and flow cytometry plots (PDF)

## Abbreviations

PSC: pluripotent stem cell
synNotch: synthetic Notch
RD: reaction-diffusion
PI: positional information
HEK: human embryonic kidney cells
hESCs: human embryonic stem cells
iPSCs: induced pluripotent stem cells
GFP: green fluorescent protein
nfGFP: nonfluorescent green fluorescent protein
MATRIX: Material Activated To Regulate Inducible gene eXpression
GBP: glycosaminoglycan binding peptide
cRGD: cyclic arginine-glycine-aspartate
mDa: midbrain dopaminergic
PEG: polyethylene glycol
WT: wild-type

## References

[ref1] Zakrzewski W.; Dobrzyński M.; Szymonowicz M.; Rybak Z. Stem cells: past, present, and future. Stem Cell Research & Therapy 2019, 10 (1), 68 10.1186/s13287-019-1165-5. 30808416 PMC6390367

[ref2] Thomson J. A.; Odorico J. S. Human embryonic stem cell and embryonic germ cell lines. Trends Biotechnol. 2000, 18 (2), 53–57. 10.1016/S0167-7799(99)01410-9. 10652509

[ref3] Watt F. M.; Hogan a. B. L. M. Out of Eden: Stem Cells and Their Niches. Science 2000, 287 (5457), 1427–1430. 10.1126/science.287.5457.1427. 10688781

[ref4] Kolind K.; Leong K. W.; Besenbacher F.; Foss M. Guidance of stem cell fate on 2D patterned surfaces. Biomaterials 2012, 33 (28), 6626–6633. 10.1016/j.biomaterials.2012.05.070. 22748769

[ref5] Pennings S.; Liu K. J.; Qian H. The Stem Cell Niche: Interactions between Stem Cells and Their Environment. Stem Cells Int. 2018, 2018, 4879379 10.1155/2018/4879379. 30405721 PMC6204189

[ref6] Peerani R.; Rao B. M.; Bauwens C.; Yin T.; Wood G. A.; Nagy A.; Kumacheva E.; Zandstra P. W. Niche-mediated control of human embryonic stem cell self-renewal and differentiation. EMBO Journal 2007, 26 (22), 4744–4755. 10.1038/sj.emboj.7601896. 17948051 PMC2080799

[ref7] Schofield R. The relationship between the spleen colony-forming cell and the haemopoietic stem cell. Blood Cells 1978, 4 (1–2), 7–25. 747780

[ref8] Maheden K.; Bashth O. S.; Shakiba N. Evening the playing field: microenvironmental control over stem cell competition during fate programming. Current Opinion in Genetics & Development 2021, 70, 66–75. 10.1016/j.gde.2021.05.008. 34153929

[ref9] Ort C.; Dayekh K.; Xing M.; Mequanint K. Emerging Strategies for Stem Cell Lineage Commitment in Tissue Engineering and Regenerative Medicine. ACS Biomaterials Science & Engineering 2018, 4 (11), 3644–3657. 10.1021/acsbiomaterials.8b00532. 33429592

[ref10] Brassard J. A.; Lutolf M. P. Engineering Stem Cell Self-organization to Build Better Organoids. Cell Stem Cell 2019, 24 (6), 860–876. 10.1016/j.stem.2019.05.005. 31173716

[ref11] Priester C.; MacDonald A.; Dhar M.; Bow A. Examining the Characteristics and Applications of Mesenchymal, Induced Pluripotent, and Embryonic Stem Cells for Tissue Engineering Approaches across the Germ Layers. Pharmaceuticals 2020, 13 (11), 344 10.3390/ph13110344. 33114710 PMC7692540

[ref12] Wolpert L. Positional information and the spatial pattern of cellular differentiation. J. Theor. Biol. 1969, 25 (1), 1–47. 10.1016/S0022-5193(69)80016-0. 4390734

[ref13] Turing A. M. The chemical basis of morphogenesis. Philos. Trans. R. Soc., B 1952, 237 (641), 37–72. 10.1098/rstb.1952.0012.

[ref14] Meinhardt H.; Gierer A. Applications of a theory of biological pattern formation based on lateral inhibition. Journal of Cell Science 1974, 15 (2), 321–346. 10.1242/jcs.15.2.321. 4859215

[ref15] Tewary M.; Ostblom J.; Prochazka L.; Zulueta-Coarasa T.; Shakiba N.; Fernandez-Gonzalez R.; Zandstra P. W. A stepwise model of reaction-diffusion and positional information governs self-organized human peri-gastrulation-like patterning. Development 2017, 144 (23), 4298–4312. 10.1242/dev.149658. 28870989 PMC5769627

[ref16] Burt M.; Bhattachaya R.; Okafor A. E.; Musah S. Guided Differentiation of Mature Kidney Podocytes from Human Induced Pluripotent Stem Cells Under Chemically Defined Conditions. J. Vis Exp 2020, (161), e61299 10.3791/61299. 32716365

[ref17] Ellis M. E.; Harris B. N.; Hashemi M.; Harvell B. J.; Bush M. Z.; Hicks E. E.; Finklea F. B.; Wang E. M.; Nataraj R.; Young N. P.; Turnbull M. D.; Lipke E. A. Human Induced Pluripotent Stem Cell Encapsulation Geometry Impacts Three-Dimensional Developing Human Engineered Cardiac Tissue Functionality. Tissue Engineering Part A 2022, 28 (23–24), 990–1000. 10.1089/ten.tea.2022.0107. 36170590 PMC9807282

[ref18] Giandomenico S. L.; Sutcliffe M.; Lancaster M. A. Generation and long-term culture of advanced cerebral organoids for studying later stages of neural development. Nat. Protoc 2021, 16 (2), 579–602. 10.1038/s41596-020-00433-w. 33328611 PMC7611064

[ref19] Polstein L. R.; Juhas M.; Hanna G.; Bursac N.; Gersbach C. A. An Engineered Optogenetic Switch for Spatiotemporal Control of Gene Expression, Cell Differentiation, and Tissue Morphogenesis. ACS Synth. Biol. 2017, 6 (11), 2003–2013. 10.1021/acssynbio.7b00147. 28793186 PMC5767923

[ref20] Repina N. A.; Johnson H. J.; Bao X.; Zimmermann J. A.; Joy D. A.; Bi S. Z.; Kane R. S.; Schaffer D. V. Optogenetic control of Wnt signaling models cell-intrinsic embryogenic patterning using 2D human pluripotent stem cell culture. Development 2023, 150 (14), dev201386 10.1242/dev.201386. 37401411 PMC10399980

[ref21] Scheller L.; Strittmatter T.; Fuchs D.; Bojar D.; Fussenegger M. Generalized extracellular molecule sensor platform for programming cellular behavior. Nat. Chem. Biol. 2018, 14 (7), 723–729. 10.1038/s41589-018-0046-z. 29686358

[ref22] Daringer N. M.; Dudek R. M.; Schwarz K. A.; Leonard J. N. Modular extracellular sensor architecture for engineering mammalian cell-based devices. ACS Synth. Biol. 2014, 3 (12), 892–902. 10.1021/sb400128g. 24611683 PMC4161666

[ref23] Pan Y.; Yoon S.; Sun J.; Huang Z.; Lee C.; Allen M.; Wu Y.; Chang Y. J.; Sadelain M.; Shung K. K.; Chien S.; Wang Y. Mechanogenetics for the remote and noninvasive control of cancer immunotherapy. Proc. Natl. Acad. Sci. U. S. A. 2018, 115 (5), 992–997. 10.1073/pnas.1714900115. 29343642 PMC5798350

[ref24] Nims R. J.; Pferdehirt L.; Ho N. B.; Savadipour A.; Lorentz J.; Sohi S.; Kassab J.; Ross A. K.; O’Conor C. J.; Liedtke W. B.; Zhang B.; McNulty A. L.; Guilak F. A synthetic mechanogenetic gene circuit for autonomous drug delivery in engineered tissues. Science Advances 2021, 7 (5), eabd9858 10.1126/sciadv.abd9858. 33571125 PMC7840132

[ref25] Toda S.; McKeithan W. L.; Hakkinen T. J.; Lopez P.; Klein O. D.; Lim W. A. Engineering synthetic morphogen systems that can program multicellular patterning. Science 2020, 370 (6514), 327–331. 10.1126/science.abc0033. 33060357 PMC7986291

[ref26] Toda S.; Frankel N. W.; Lim W. A. Engineering cell–cell communication networks: programming multicellular behaviors. Curr. Opin. Chem. Biol. 2019, 52, 31–38. 10.1016/j.cbpa.2019.04.020. 31150899

[ref27] Ebrahimkhani M. R.; Ebisuya M. Synthetic developmental biology: build and control multicellular systems. Curr. Opin. Chem. Biol. 2019, 52, 9–15. 10.1016/j.cbpa.2019.04.006. 31102790

[ref28] Sekine R.; Shibata T.; Ebisuya M. Synthetic mammalian pattern formation driven by differential diffusivity of Nodal and Lefty. Nat. Commun. 2018, 9 (1), 5456 10.1038/s41467-018-07847-x. 30575724 PMC6303393

[ref29] Glykofrydis F.; Cachat E.; Berzanskyte I.; Dzierzak E.; Davies J. A. Bioengineering Self-Organizing Signaling Centers to Control Embryoid Body Pattern Elaboration. ACS Synth. Biol. 2021, 10 (6), 1465–1480. 10.1021/acssynbio.1c00060. 34019395

[ref30] Morsut L.; Roybal K. T.; Xiong X.; Gordley R. M.; Coyle S. M.; Thomson M.; Lim W. A. Engineering Customized Cell Sensing and Response Behaviors Using Synthetic Notch Receptors. Cell 2016, 164 (4), 780–791. 10.1016/j.cell.2016.01.012. 26830878 PMC4752866

[ref31] Gordon W. R.; Zimmerman B.; He L.; Miles L. J.; Huang J.; Tiyanont K.; McArthur D. G.; Aster J. C.; Perrimon N.; Loparo J. J.; Blacklow S. C. Mechanical allostery: evidence for a force requirement in the proteolytic activation of Notch. Dev. Cell 2015, 33 (6), 729–736. 10.1016/j.devcel.2015.05.004. 26051539 PMC4481192

[ref32] Garibyan M.; Hoffman T.; Makaske T.; Do S. K.; Wu Y.; Williams B. A.; March A. R.; Cho N.; Pedroncelli N.; Lima R. E.; Soto J.; Jackson B.; Santoso J. W.; Khademhosseini A.; Thomson M.; Li S.; McCain M. L.; Morsut L. Engineering programmable material-to-cell pathways via synthetic notch receptors to spatially control differentiation in multicellular constructs. Nat. Commun. 2024, 15, 5891 10.1038/s41467-024-50126-1. 39003263 PMC11246427

[ref33] Stapornwongkul K. S.; de Gennes M.; Cocconi L.; Salbreux G.; Vincent J.-P. Patterning and growth control in vivo by an engineered GFP gradient. Science 2020, 370 (6514), 321–327. 10.1126/science.abb8205. 33060356 PMC7611032

[ref34] Matsuda M.; Koga M.; Woltjen K.; Nishida E.; Ebisuya M. Synthetic lateral inhibition governs cell-type bifurcation with robust ratios. Nat. Commun. 2015, 6 (1), 6195 10.1038/ncomms7195. 25652697

[ref35] Cachat E.; Liu W.; Martin K. C.; Yuan X.; Yin H.; Hohenstein P.; Davies J. A. 2- and 3-dimensional synthetic large-scale de novo patterning by mammalian cells through phase separation. Sci. Rep 2016, 6, 20664 10.1038/srep20664. 26857385 PMC4746622

[ref36] Lee J. C.; Brien H. J.; Walton B. L.; Eidman Z. M.; Toda S.; Lim W. A.; Brunger J. M. Instructional materials that control cellular activity through synthetic Notch receptors. Biomaterials 2023, 297, 122099 10.1016/j.biomaterials.2023.122099. 37023529 PMC10320837

[ref37] Deglincerti A.; Etoc F.; Guerra M. C.; Martyn I.; Metzger J.; Ruzo A.; Simunovic M.; Yoney A.; Brivanlou A. H.; Siggia E.; Warmflash A. Self-organization of human embryonic stem cells on micropatterns. Nat. Protoc. 2016, 11 (11), 2223–2232. 10.1038/nprot.2016.131. 27735934 PMC5821517

[ref38] Kim T. W.; Piao J.; Koo S. Y.; Kriks S.; Chung S. Y.; Betel D.; Socci N. D.; Choi S. J.; Zabierowski S.; Dubose B. N.; Hill E. J.; Mosharov E. V.; Irion S.; Tomishima M. J.; Tabar V.; Studer L. Biphasic Activation of WNT Signaling Facilitates the Derivation of Midbrain Dopamine Neurons from hESCs for Translational Use. Cell Stem Cell 2021, 28 (2), 343–355. 10.1016/j.stem.2021.01.005. 33545081 PMC8006469

[ref39] Martyn I.; Kanno T. Y.; Ruzo A.; Siggia E. D.; Brivanlou A. H. Self-organization of a human organizer by combined Wnt and Nodal signalling. Nature 2018, 558 (7708), 132–135. 10.1038/s41586-018-0150-y. 29795348 PMC6077985

[ref40] Fridy P. C.; Li Y.; Keegan S.; Thompson M. K.; Nudelman I.; Scheid J. F.; Oeffinger M.; Nussenzweig M. C.; Fenyö D.; Chait B. T.; Rout M. P. A robust pipeline for rapid production of versatile nanobody repertoires. Nat. Methods 2014, 11 (12), 1253–1260. 10.1038/nmeth.3170. 25362362 PMC4272012

[ref41] Kirchhofer A.; Helma J.; Schmidthals K.; Frauer C.; Cui S.; Karcher A.; Pellis M.; Muyldermans S.; Casas-Delucchi C. S.; Cardoso M. C.; Leonhardt H.; Hopfner K. P.; Rothbauer U. Modulation of protein properties in living cells using nanobodies. Nature Structural & Molecular Biology 2010, 17 (1), 133–138. 10.1038/nsmb.1727. 20010839

[ref42] Kowarz E.; Löscher D.; Marschalek R. Optimized Sleeping Beauty transposons rapidly generate stable transgenic cell lines. Biotechnology Journal 2015, 10 (4), 647–653. 10.1002/biot.201400821. 25650551

[ref43] Kumar K. K.; Lowe E. W.; Aboud A. A.; Neely M. D.; Redha R.; Bauer J. A.; Odak M.; Weaver C. D.; Meiler J.; Aschner M.; Bowman A. B. Cellular manganese content is developmentally regulated in human dopaminergic neurons. Sci. Rep 2014, 4, 6801 10.1038/srep06801. 25348053 PMC4210885

[ref44] Hollmann E. K.; Bailey A. K.; Potharazu A. V.; Neely M. D.; Bowman A. B.; Lippmann E. S. Accelerated differentiation of human induced pluripotent stem cells to blood–brain barrier endothelial cells. Fluids and Barriers of the CNS 2017, 14 (1), 9 10.1186/s12987-017-0059-0. 28407791 PMC5390351

[ref45] Pantazis C. B.; Yang A.; Lara E.; McDonough J. A.; Blauwendraat C.; Peng L.; Oguro H.; Kanaujiya J.; Zou J.; Sebesta D.; Pratt G.; Cross E.; Blockwick J.; Buxton P.; Kinner-Bibeau L.; Medura C.; Tompkins C.; Hughes S.; Santiana M.; Faghri F.; Nalls M. A.; Vitale D.; Ballard S.; Qi Y. A.; Ramos D. M.; Anderson K. M.; Stadler J.; Narayan P.; Papademetriou J.; Reilly L.; Nelson M. P.; Aggarwal S.; Rosen L. U.; Kirwan P.; Pisupati V.; Coon S. L.; Scholz S. W.; Priebe T.; Öttl M.; Dong J.; Meijer M.; Janssen L. J. M.; Lourenco V. S.; van der Kant R.; Crusius D.; Paquet D.; Raulin A. C.; Bu G.; Held A.; Wainger B. J.; Gabriele R. M. C.; Casey J. M.; Wray S.; Abu-Bonsrah D.; Parish C. L.; Beccari M. S.; Cleveland D. W.; Li E.; Rose I. V. L.; Kampmann M.; Calatayud Aristoy C.; Verstreken P.; Heinrich L.; Chen M. Y.; Schüle B.; Dou D.; Holzbaur E. L. F.; Zanellati M. C.; Basundra R.; Deshmukh M.; Cohen S.; Khanna R.; Raman M.; Nevin Z. S.; Matia M.; Van Lent J.; Timmerman V.; Conklin B. R.; Johnson Chase K.; Zhang K.; Funes S.; Bosco D. A.; Erlebach L.; Welzer M.; Kronenberg-Versteeg D.; Lyu G.; Arenas E.; Coccia E.; Sarrafha L.; Ahfeldt T.; Marioni J. C.; Skarnes W. C.; Cookson M. R.; Ward M. E.; Merkle F. T. A reference human induced pluripotent stem cell line for large-scale collaborative studies. Cell Stem Cell. 2022, 29 (12), 1685–1702.e22. 10.1016/j.stem.2022.11.004. 36459969 PMC9782786

[ref46] Mátés L.; Chuah M. K. L.; Belay E.; Jerchow B.; Manoj N.; Acosta-Sanchez A.; Grzela D. P.; Schmitt A.; Becker K.; Matrai J.; Ma L.; Samara-Kuko E.; Gysemans C.; Pryputniewicz D.; Miskey C.; Fletcher B.; VandenDriessche T.; Ivics Z.; Izsvák Molecular evolution of a novel hyperactive Sleeping Beauty transposase enables robust stable gene transfer in vertebrates. Nat. Genet. 2009, 41 (6), 753–761. 10.1038/ng.343. 19412179

[ref47] Battad J. M.; Traore D. A. K.; Byres E.; Rossjohn J.; Devenish R. J.; Olsen S.; Wilce M. C. J.; Prescott M. A Green Fluorescent Protein Containing a QFG Tri-Peptide Chromophore: Optical Properties and X-Ray Crystal Structure. PLoS One 2012, 7 (10), e47331 10.1371/journal.pone.0047331. 23071789 PMC3468514

[ref48] Chen G.; Gulbranson D. R.; Hou Z.; Bolin J. M.; Ruotti V.; Probasco M. D.; Smuga-Otto K.; Howden S. E.; Diol N. R.; Propson N. E.; Wagner R.; Lee G. O.; Antosiewicz-Bourget J.; Teng J. M. C.; Thomson J. A. Chemically defined conditions for human iPSC derivation and culture. Nat. Methods 2011, 8 (5), 424–429. 10.1038/nmeth.1593. 21478862 PMC3084903

[ref49] Stossi F.; Singh P. K. Basic Image Analysis and Manipulation in ImageJ/Fiji. Current Protocols 2023, 3 (7), e849 10.1002/cpz1.849. 37498127

[ref50] Warmflash A.; Sorre B.; Etoc F.; Siggia E. D.; Brivanlou A. H. A method to recapitulate early embryonic spatial patterning in human embryonic stem cells. Nat. Methods 2014, 11 (8), 847–854. 10.1038/nmeth.3016. 24973948 PMC4341966

[ref51] Faial T.; Bernardo A. S.; Mendjan S.; Diamanti E.; Ortmann D.; Gentsch G. E.; Mascetti V. L.; Trotter M. W.; Smith J. C.; Pedersen R. A. Brachyury and SMAD signalling collaboratively orchestrate distinct mesoderm and endoderm gene regulatory networks in differentiating human embryonic stem cells. Development 2015, 142 (12), 2121–2135. 10.1242/dev.117838. 26015544 PMC4483767

[ref52] Hiraoka-Kanie M.; Miyagishi M.; Yamashita J. K. Differentiation stage-specific analysis of gene function with inducible short hair-pin RNA in differentiating embryonic stem cells. Biochem. Biophys. Res. Commun. 2006, 351 (3), 669–674. 10.1016/j.bbrc.2006.10.108. 17084387

[ref53] Xu P.; He H.; Gao Q.; Zhou Y.; Wu Z.; Zhang X.; Sun L.; Hu G.; Guan Q.; You Z.; Zhang X.; Zheng W.; Xiong M.; Chen Y. Human midbrain dopaminergic neuronal differentiation markers predict cell therapy outcomes in a Parkinson’s disease model. J. Clin Invest 2022, 132 (14), 1–18. 10.1172/JCI156768. PMC9282930 35700056

[ref54] Kriks S.; Shim J. W.; Piao J.; Ganat Y. M.; Wakeman D. R.; Xie Z.; Carrillo-Reid L.; Auyeung G.; Antonacci C.; Buch A.; Yang L.; Beal M. F.; Surmeier D. J.; Kordower J. H.; Tabar V.; Studer L. Dopamine neurons derived from human ES cells efficiently engraft in animal models of Parkinson’s disease. Nature 2011, 480 (7378), 547–551. 10.1038/nature10648. 22056989 PMC3245796

[ref55] Xi R. Anchoring stem cells in the niche by cell adhesion molecules. Cell Adh Migr 2009, 3 (4), 396–401. 10.4161/cam.3.4.8604. 19421010 PMC2802755

[ref56] Chen S.; Lewallen M.; Xie T. Adhesion in the stem cell niche: biological roles and regulation. Development 2013, 140 (2), 255–265. 10.1242/dev.083139. 23250203 PMC3597204

[ref57] Meng Y.; Eshghi S.; Li Y. J.; Schmidt R.; Schaffer D. V.; Healy K. E. Characterization of integrin engagement during defined human embryonic stem cell culture. FASEB J. 2010, 24 (4), 1056–1065. 10.1096/fj.08-126821. 19933311 PMC2845424

[ref58] Song X.; Zhu C. H.; Doan C.; Xie T. Germline stem cells anchored by adherens junctions in the Drosophila ovary niches. Science 2002, 296 (5574), 1855–1857. 10.1126/science.1069871. 12052957

[ref59] Tanentzapf G.; Devenport D.; Godt D.; Brown N. H. Integrin-dependent anchoring of a stem-cell niche. Nat. Cell Biol. 2007, 9 (12), 1413–1418. 10.1038/ncb1660. 17982446 PMC3529653

[ref60] Klim J. R.; Li L.; Wrighton P. J.; Piekarczyk M. S.; Kiessling L. L. A defined glycosaminoglycan-binding substratum for human pluripotent stem cells. Nat. Methods 2010, 7 (12), 989–994. 10.1038/nmeth.1532. 21076418 PMC2994976

[ref61] Wrighton P. J.; Klim J. R.; Hernandez B. A.; Koonce C. H.; Kamp T. J.; Kiessling L. L. Signals from the surface modulate differentiation of human pluripotent stem cells through glycosaminoglycans and integrins. Proc. Natl. Acad. Sci. U. S. A. 2014, 111 (51), 18126–18131. 10.1073/pnas.1409525111. 25422477 PMC4280649

[ref62] Kolhar P.; Kotamraju V. R.; Hikita S. T.; Clegg D. O.; Ruoslahti E. Synthetic surfaces for human embryonic stem cell culture. J. Biotechnol. 2010, 146 (3), 143–146. 10.1016/j.jbiotec.2010.01.016. 20132848

[ref63] Hersel U.; Dahmen C.; Kessler H. RGD modified polymers: biomaterials for stimulated cell adhesion and beyond. Biomaterials 2003, 24 (24), 4385–4415. 10.1016/S0142-9612(03)00343-0. 12922151

[ref64] Jeschke B.; Meyer J.; Jonczyk A.; Kessler H.; Adamietz P.; Meenen N. M.; Kantlehner M.; Goepfert C.; Nies B. RGD-peptides for tissue engineering of articular cartilage. Biomaterials 2002, 23 (16), 3455–3463. 10.1016/S0142-9612(02)00052-2. 12099289

[ref65] Indana D.; Agarwal P.; Bhutani N.; Chaudhuri O. Viscoelasticity and Adhesion Signaling in Biomaterials Control Human Pluripotent Stem Cell Morphogenesis in 3D Culture. Adv. Mater. 2021, 33 (43), 2101966 10.1002/adma.202101966. 34499389

[ref66] Afewerki S.; Sheikhi A.; Kannan S.; Ahadian S.; Khademhosseini A. Gelatin-polysaccharide composite scaffolds for 3D cell culture and tissue engineering: Towards natural therapeutics. Bioengineering & Translational Medicine 2019, 4 (1), 96–115. 10.1002/btm2.10124. 30680322 PMC6336672

[ref67] Chhabra S.; Liu L.; Goh R.; Kong X.; Warmflash A. Dissecting the dynamics of signaling events in the BMP, WNT, and NODAL cascade during self-organized fate patterning in human gastruloids. PLoS Biol. 2019, 17 (10), e3000498 10.1371/journal.pbio.3000498. 31613879 PMC6814242

[ref68] Martyn I.; Brivanlou A. H.; Siggia E. D. A wave of WNT signaling balanced by secreted inhibitors controls primitive streak formation in micropattern colonies of human embryonic stem cells. Development 2019, 146 (6), dev172791 10.1242/dev.172791. 30814117 PMC6451321

[ref69] Prochazka L.; Michaels Y. S.; Lau C.; Jones R. D.; Siu M.; Yin T.; Wu D.; Jang E.; Vázquez-Cantú M.; Gilbert P. M.; Kaul H.; Benenson Y.; Zandstra P. W. Synthetic gene circuits for cell state detection and protein tuning in human pluripotent stem cells. Molecular Systems Biology 2022, 18 (11), e10886 10.15252/msb.202110886. 36366891 PMC9650275

[ref70] Basson M. A. Signaling in cell differentiation and morphogenesis. Cold Spring Harb Perspect Biol. 2012, 4 (6), a008151 10.1101/cshperspect.a008151. 22570373 PMC3367549

[ref71] Lewis J. From Signals to Patterns: Space, Time, and Mathematics in Developmental Biology. Science 2008, 322 (5900), 399–403. 10.1126/science.1166154. 18927385

[ref72] Ben-Haim N.; Lu C.; Guzman-Ayala M.; Pescatore L.; Mesnard D.; Bischofberger M.; Naef F.; Robertson E. J.; Constam D. B. The nodal precursor acting via activin receptors induces mesoderm by maintaining a source of its convertases and BMP4. Dev Cell 2006, 11 (3), 313–323. 10.1016/j.devcel.2006.07.005. 16950123

[ref73] Rivera-Pérez J. A.; Magnuson T. Primitive streak formation in mice is preceded by localized activation of Brachyury and Wnt3. Dev. Biol. 2005, 288 (2), 363–371. 10.1016/j.ydbio.2005.09.012. 16289026

[ref74] Zhao W.; Ji X.; Zhang F.; Li L.; Ma L. Embryonic stem cell markers. Molecules 2012, 17 (6), 6196–6236. 10.3390/molecules17066196. 22634835 PMC6268870

[ref75] Li S.; Huang Q.; Mao J.; LI Q. FGF signaling mediates definitive endoderm formation by regulating epithelial-to-mesenchymal transition and cell proliferation. Int. J. Dev Biol. 2020, 64 (10–11–12), 471–477. 10.1387/ijdb.190372ql. 33336709

[ref76] Coolen M.; Nicolle D.; Plouhinec J.-L.; Gombault A.; Sauka-Spengler T.; Menuet A.; Pieau C.; Mazan S. Molecular Characterization of the Gastrula in the Turtle Emys orbicularis: An Evolutionary Perspective on Gastrulation. PLoS One 2008, 3 (7), e2676 10.1371/journal.pone.0002676. 18628985 PMC2442194

[ref77] Mattes B.; Weber S.; Peres J.; Chen Q.; Davidson G.; Houart C.; Scholpp S. Wnt3 and Wnt3a are required for induction of the mid-diencephalic organizer in the caudal forebrain. Neural Development 2012, 7 (1), 12 10.1186/1749-8104-7-12. 22475147 PMC3349543

[ref78] Joksimovic M.; Awatramani R. Wnt/β-catenin signaling in midbrain dopaminergic neuron specification and neurogenesis. Journal of Molecular Cell Biology 2014, 6 (1), 27–33. 10.1093/jmcb/mjt043. 24287202

[ref79] Castelo-Branco G.; Wagner J.; Rodriguez F. J.; Kele J.; Sousa K.; Rawal N.; Pasolli H. A.; Fuchs E.; Kitajewski J.; Arenas E. Differential regulation of midbrain dopaminergic neuron development by Wnt-1, Wnt-3a, and Wnt-5a. Proc. Natl. Acad. Sci. U. S. A. 2003, 100 (22), 12747–52. 10.1073/pnas.1534900100. 14557550 PMC240689

[ref80] Chung C. Y.; Licznerski P.; Alavian K. N.; Simeone A.; Lin Z.; Martin E.; Vance J.; Isacson O. The transcription factor orthodenticle homeobox 2 influences axonal projections and vulnerability of midbrain dopaminergic neurons. Brain 2010, 133 (7), 2022–2031. 10.1093/brain/awq142. 20573704 PMC2892944

[ref81] Dessaud E.; Yang L. L.; Hill K.; Cox B.; Ulloa F.; Ribeiro A.; Mynett A.; Novitch B. G.; Briscoe J. Interpretation of the sonic hedgehog morphogen gradient by a temporal adaptation mechanism. Nature 2007, 450 (7170), 717–720. 10.1038/nature06347. 18046410

[ref82] Pagès F.; Kerridge S. Morphogen gradients: a question of time or concentration?. Trends in Genetics 2000, 16 (1), 40–44. 10.1016/S0168-9525(99)01880-6. 10637630

[ref83] Heemskerk I.; Burt K.; Miller M.; Chhabra S.; Guerra M. C.; Liu L.; Warmflash A. Rapid changes in morphogen concentration control self-organized patterning in human embryonic stem cells. eLife 2019, 8, e40526 10.7554/eLife.40526. 30829572 PMC6398983

[ref84] Freeman F. E.; Pitacco P.; van Dommelen L. H. A.; Nulty J.; Browe D. C.; Shin J.-Y.; Alsberg E.; Kelly D. J. 3D bioprinting spatiotemporally defined patterns of growth factors to tightly control tissue regeneration. Science Advances 2020, 6 (33), eabb5093 10.1126/sciadv.abb5093. 32851179 PMC7428335

[ref85] Greber D.; Fussenegger M. An engineered mammalian band-pass network. Nucleic Acids Res. 2010, 38 (18), e174–e174. 10.1093/nar/gkq671. 20693530 PMC2952875

[ref86] Wang X.; Liang Q.; Luo Y.; Ye J.; Yu Y.; Chen F. Engineering the next generation of theranostic biomaterials with synthetic biology. Bioactive Materials 2024, 32, 514–529. 10.1016/j.bioactmat.2023.10.018. 38026437 PMC10660023

[ref87] Li P.; Markson J. S.; Wang S.; Chen S.; Vachharajani V.; Elowitz M. B. Morphogen gradient reconstitution reveals Hedgehog pathway design principles. Science 2018, 360 (6388), 543–548. 10.1126/science.aao0645. 29622726 PMC6516753

[ref88] Miki K.; Endo K.; Takahashi S.; Funakoshi S.; Takei I.; Katayama S.; Toyoda T.; Kotaka M.; Takaki T.; Umeda M.; Okubo C.; Nishikawa M.; Oishi A.; Narita M.; Miyashita I.; Asano K.; Hayashi K.; Osafune K.; Yamanaka S.; Saito H.; Yoshida Y. Efficient Detection and Purification of Cell Populations Using Synthetic MicroRNA Switches. Cell Stem Cell 2015, 16 (6), 699–711. 10.1016/j.stem.2015.04.005. 26004781

[ref89] De Santis R.; Etoc F.; Rosado-Olivieri E. A.; Brivanlou A. H. Self-organization of human dorsal-ventral forebrain structures by light induced SHH. Nat. Commun. 2021, 12 (1), 6768 10.1038/s41467-021-26881-w. 34799555 PMC8604999

[ref90] Miyamoto Y.; Sakane F.; Hashimoto K. N-cadherin-based adherens junction regulates the maintenance, proliferation, and differentiation of neural progenitor cells during development. Cell Adh Migr 2015, 9 (3), 183–192. 10.1080/19336918.2015.1005466. 25869655 PMC4594476

[ref91] Punovuori K.; Migueles R. P.; Malaguti M.; Blin G.; Macleod K. G.; Carragher N. O.; Pieters T.; van Roy F.; Stemmler M. P.; Lowell S. N-cadherin stabilises neural identity by dampening anti-neural signals. Development 2019, 146 (21), dev183269 10.1242/dev.183269. 31601548 PMC6857587

[ref92] O’Grady B. J.; Balotin K. M.; Bosworth A. M.; McClatchey P. M.; Weinstein R. M.; Gupta M.; Poole K. S.; Bellan L. M.; Lippmann E. S. Development of an N-Cadherin Biofunctionalized Hydrogel to Support the Formation of Synaptically Connected Neural Networks. ACS Biomater Sci. Eng. 2020, 6 (10), 5811–5822. 10.1021/acsbiomaterials.0c00885. 33320550 PMC7791574

[ref93] Lim H. J.; Mosley M. C.; Kurosu Y.; Smith Callahan L. A. Concentration dependent survival and neural differentiation of murine embryonic stem cells cultured on polyethylene glycol dimethacrylate hydrogels possessing a continuous concentration gradient of n-cadherin derived peptide His-Ala-Val-Asp-Lle. Acta Biomaterialia 2017, 56, 153–160. 10.1016/j.actbio.2016.11.063. 27915022

