# Supplementary material for: Templated Pluripotent Stem Cell Differentiation via Substratum-Guided Artificial Signaling
Source: ACS Biomater Sci Eng. 2024 Oct 1;10(10):6465–82. doi: 10.1021/acsbiomaterials.4c00885 (PMC11480943; doi:10.1021/acsbiomaterials.4c00885)
Supplement: Supplementary file 1 — ab4c00885_si_001.pdf [file ab4c00885_si_001.pdf]

Supplemental Information: Templated Pluripotent Stem Cell Differentiation via Substratum-Guided Artificial Signaling

Hannah J. Brien[1], Joanne C. Lee[1], Jhanvi Sharma[1], Catherine A. Hamann[1], Madeline R. Spetz[1], Ethan S. Lippmann[2,3], and Jonathan M. Brunger[1,3]*

[1]Department of Biomedical Engineering, Vanderbilt University, Nashville, Tennessee 37235, United States. [2]Department of Chemical and Biomolecular Engineering, Vanderbilt University, Nashville, Tennessee 37235, United States. [3]Center for Stem Cell Biology, Vanderbilt University, Nashville, Tennessee 37235, United States.

# of Pages: 7

# of Figures: 5

# of Tables: 1

Supplemental Table 1: Antibody Information

Antibody	Dilution	Manufacturer	Catalog #
Myc Tag (9B11) Mouse mAb (Alexa Fluor 647 Conjugate)	1:50	Cell Signaling Technology	#2233S
Alexa Fluor 647 Mouse anti-SSEA-4	1:10	BD Biosciences	560796
Alexa Fluor 555 Mouse anti-Human TRA-1-81	1:10	BD Biosciences	560123
Goat anti-human brachyury	1:200	R&D Systems	#AF2085
Rabbit anti-human VEGFR2	1:500	Cell Signaling Technology	#9698S
Sox2 (D6D9) XP Rabbit mAb	1:400	Cell Signaling Technology	#3579S
Human SOX17 antibody (polyclonal goat IgG)	1:200	R&D Systems	#AF1924
Rabbit polyclonal antibody for the C-terminal of LMX1a	1:50	Abcam	#ab139726
Goat anti-human polyclonal antibody for HNF-3 beta/FoxA2	1:40	Fisher Scientific	#AF2400
OTX2 mouse Monoclonal Antibody (1H12C4B5)	1:200	Thermo Scientific	#MA5-15854
EN1 rabbit Polyclonal Antibody	1:25	Thermo Scientific	#PA5-14149
Alexa Fluor 488-conjugated Donkey anti-rabbit IgG	1:500	Thermo Scientific	#A21206
Alexa Fluor 488-conjugated Donkey anti-mouse IgG	1:500	Thermo Scientific	#A21202
Alexa Fluor 555-conjugated Donkey anti-goat IgG	1:500	Themo Scientific	#A21432
Alexa Fluor 647-conjugated Donkey anti-goat IgG	1:500	Thermo Scientific	#A21447
Alexa Fluor 647-conjugated Donkey anti-rabbit IgG	1:500	Thermo Scientific	#A31573

DAPI (4',6-diamidino-2-phenylindole, dihydrochloride)	1:1000	Thermo Scientific	#62247

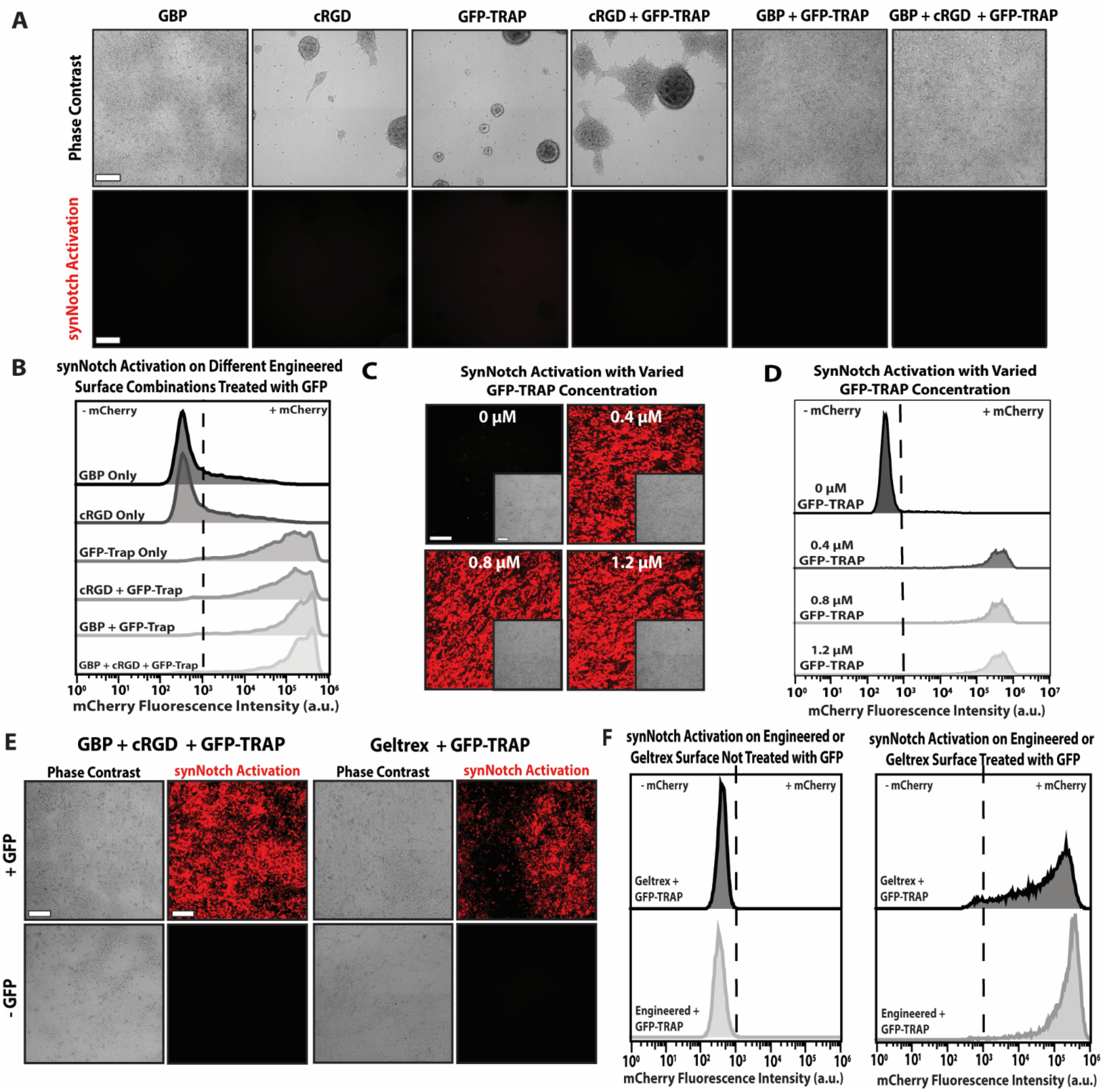

Supplemental Figure 1: Comparison of single-, dual-, and tri-peptide engineered surfaces as well as Geltrex vs. engineered surface, related to Figure 1. A) Representative phase contrast and fluorescence microscopy images of GFP-responsive reporter-synNotch H9 hESCs that were not treated with GFP. Images are from four days after plating cells on various substrata. Single peptide substrata were composed of 5 µM GBP, 5 µM cRGD, or 0.8 µM GFP-TRAP. Dual peptide surfaces were composed of 5 µM GBP or cRGD and 0.4 µM GFP-TRAP. The tri-peptide substratum was composed of 5 µM GBP, 2.15 µM cRGD, and 0.4 µM GFP-TRAP. B) Representative flow cytometry histogram of mCherry expression in synNotch-hESCs plated on various substrata and treated with 5 nM GFP. C) Representative fluorescence and phase contrast images of GFP-responsive reporter-synNotch H9 hESCs

dosed with 5 nM GFP on the tri-peptide substratum with 0, 0.4, 0.8, or 1.2 µM GFP-TRAP on day 2 of culture. D) Representative flow cytometry histograms of GFP-responsive reporter-synNotch H9 hESCs plated on surfaces with various concentrations of GFP-TRAP. E) Representative images of reporter-synNotch H9 hESCs plated on the engineered, tri-peptide substratum or a Geltrex-coated surface mixed with GFP-TRAP with and without 5 nM GFP supplementation. F) Representative flow cytometry histograms of the tri-peptide substratum and Geltrex-coated surface mixed with GFP-TRAP with and without 5 nM GFP supplementation. Scale bars = 200 µm.

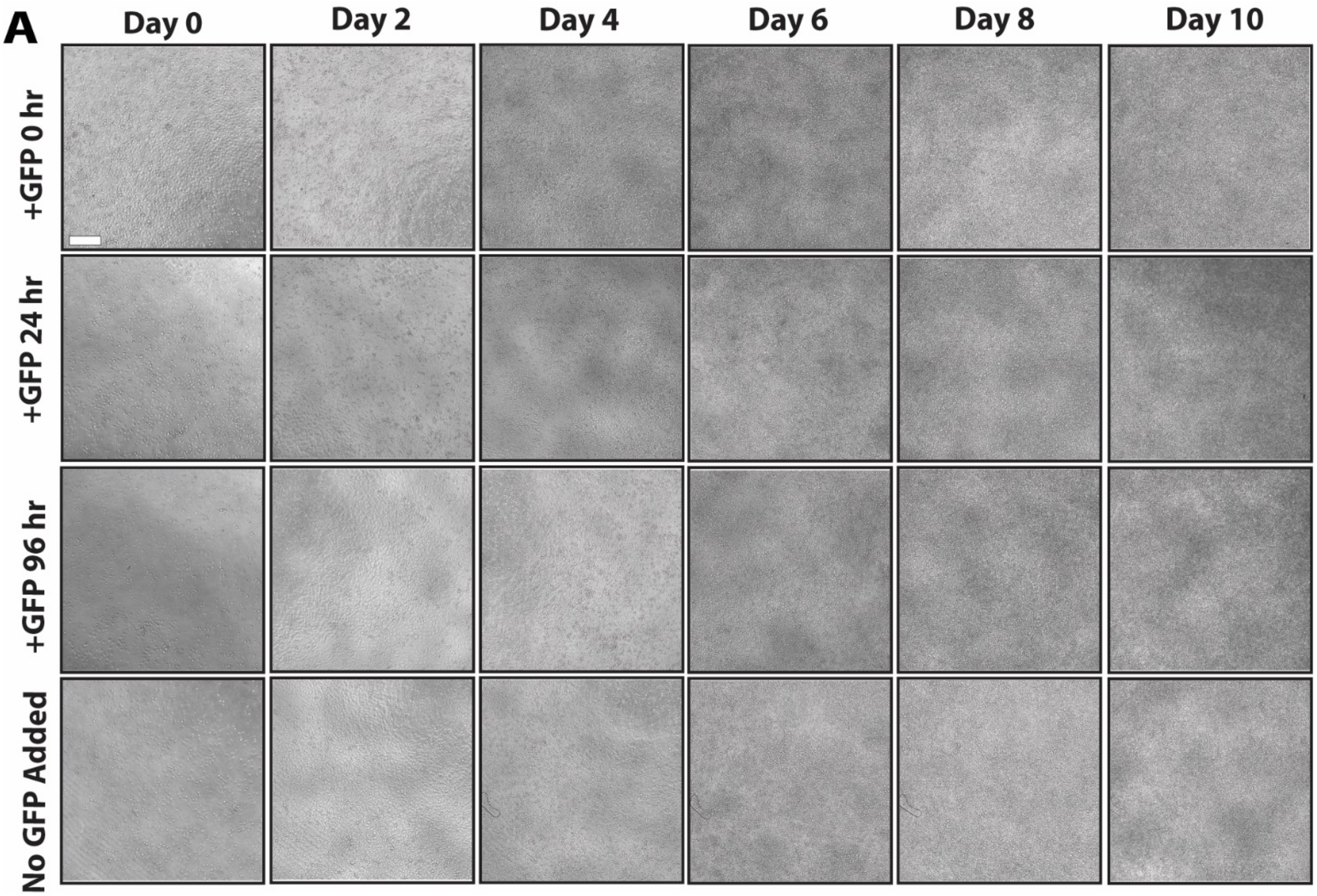

Supplemental Figure 2: Corresponding representative phase contrast images to Figure 3 of GFP-responsive reporter-synNotch H9 hESCs on the tri-peptide substratum dosed with 5 nM GFP at 0, 24, or 96 hours after plating compared to synNotch-PSCs plated on the surface without GFP. Cells remain adhered in all conditions for up to 10 days in mTeSR Plus + 10 µM ROCK inhibitor medium. Scale bar = 200 µm.

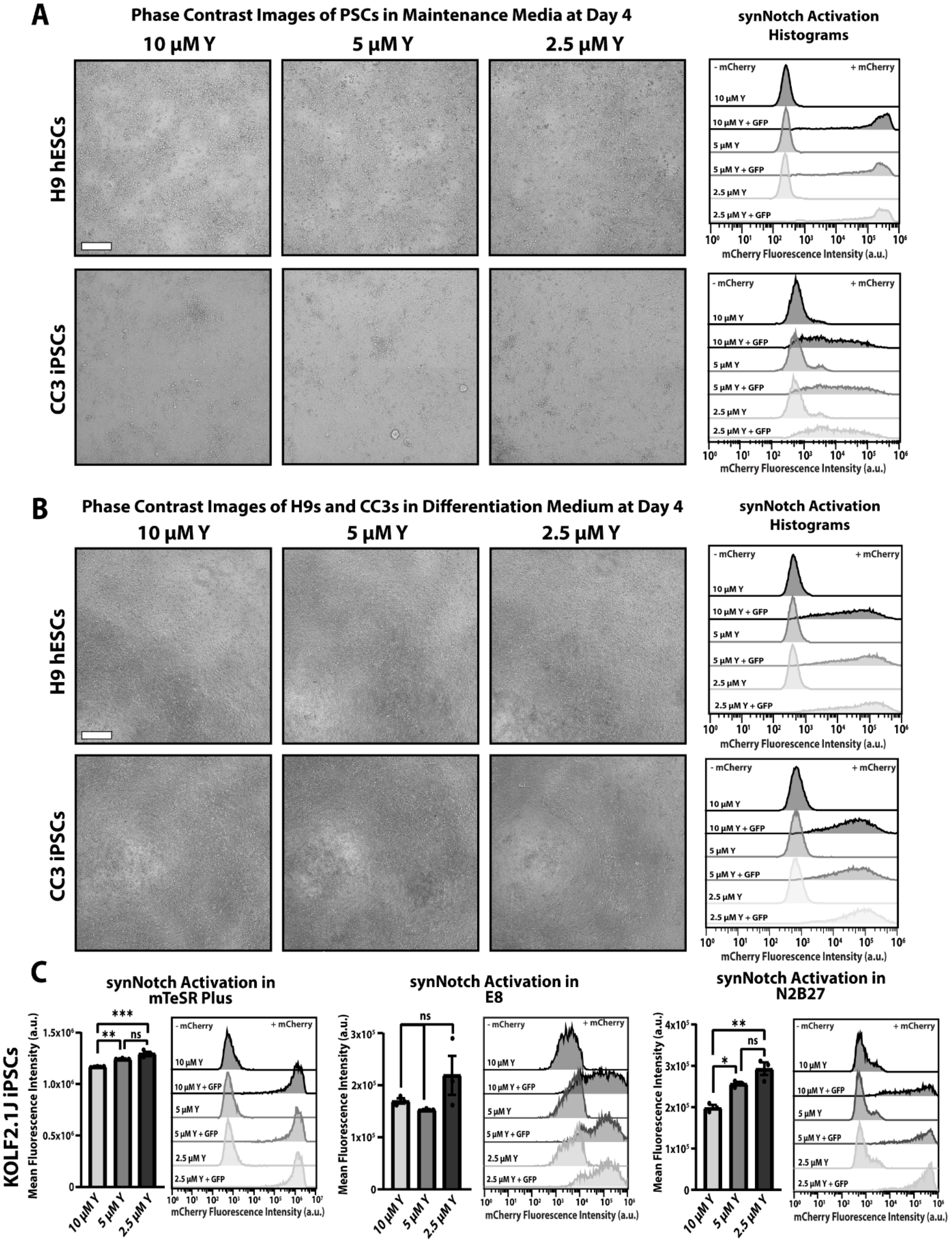

Supplemental Figure 3: Corresponding phase contrast images and flow cytometry histograms to Figure 4 of GFP-responsive synNotch H9 hESCs and CC3 iPSCs plated on the engineered substratum and maintained in A) stem cell maintenance media - mTeSR Plus for H9 hESCs and Essential 8 (E8) for CC3 iPSCs - or B) N2B27 differentiation medium supplemented with 10 µM, 5 µM, or 2.5 µM ROCK inhibitor (Y-27632) for four days. Representative flow cytometry histograms of mCherry fluorescence intensity for each media and ROCK inhibitor

concentration treated with or without 5 nM GFP are to the right of the corresponding microscopy. C) Mean mCherry fluorescence intensity of GFP-responsive reporter-synNotch KOLF2.1J iPSCs plated at a high density on the engineered substratum and maintained in stem cell maintenance medium, mTeSR Plus and Essential 8 (E8), or N2B27 differentiation medium supplemented with 10 µM, 5 µM, or 2.5 µM ROCK inhibitor (Y-27632) for four days. One-way ANOVA with Tukey's multiple comparisons post hoc: $*p < 0.05$, $**p<0.01$, $***p<0.001$. In all plots, n=3 replicate wells; error bars indicate SEM. Scale bars = 200 µm.

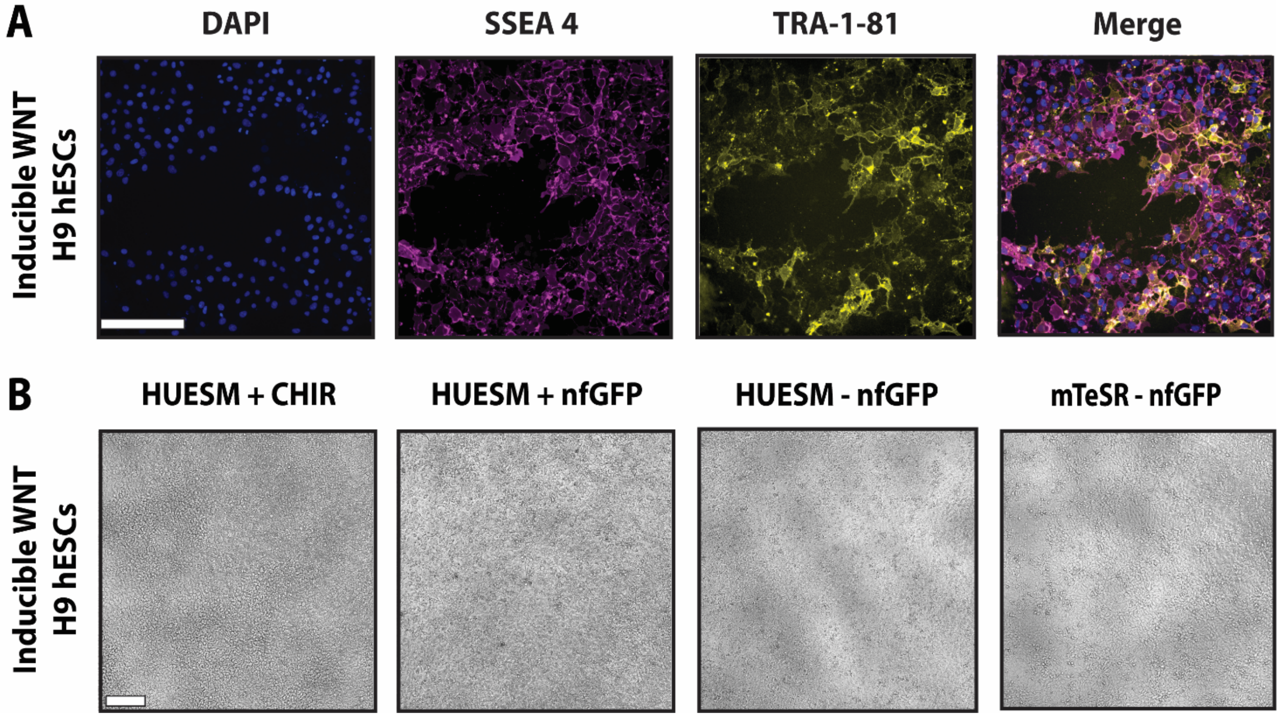

Supplemental Figure 4: A) Representative immunofluorescence images of the WNT3a-mCherry synNotch-hESCs for the pluripotency markers stage-specific embryonic antigen-4 (SSEA 4) and TRA-1-81 (Podocalyxin) in maintenance conditions, prior to initiating differentiation protocols. Wells were counterstained for nuclei with DAPI. B) Representative phase contrast microscopy images of the WNT3a-mCherry synNotch-hESCs in all four culture conditions after 48-hours on the tri-peptide substratum. Scale bars = 200 µm.

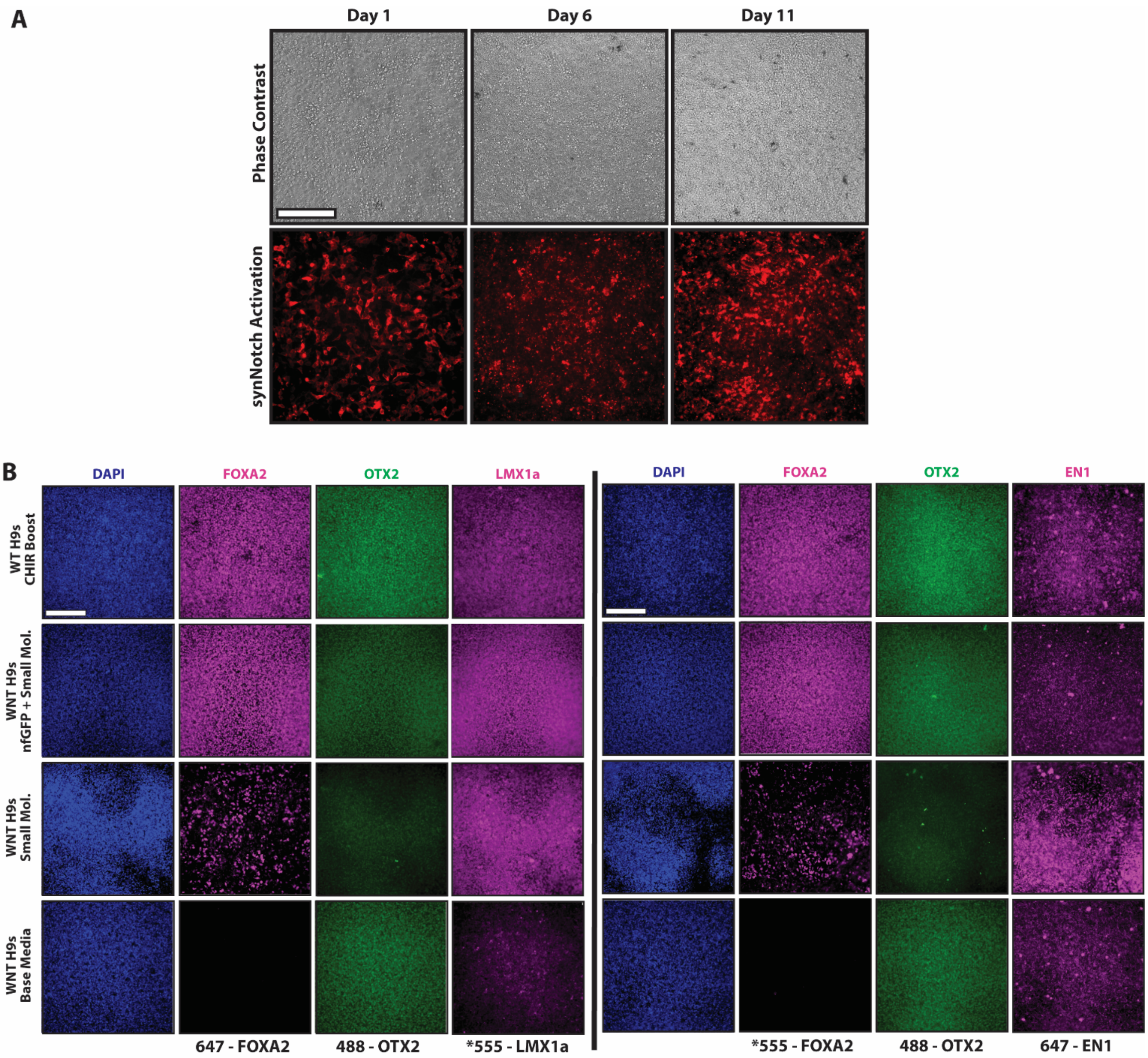

Supplemental Figure 5: Material-mediated mDA progenitor differentiation via the PSC-MATRIX platform, related to Figure 7. A) Representative phase contrast and fluorescence microscopy of inducible WNT3a synNotch-hESCs from Figure 7 depicts ligand-dependent synNotch activation of mCherry expression, which is co-expressed with WNT3a, at day 1, 6, and 11 of the mDa neuron differentiation. Furthermore, cells remained adhered to the tri-peptide surface for the duration of the 11-day protocol. B) Additional immunofluorescence images for FOXA2, OTX2, LMX1a, and EN1 expression on day 11. Images represent separate replicates of independently immunolabeled samples as compared to those shown in Figure 7. A subset of these wells made use of different secondary antibodies. Fluorophores associated with secondary antibodies used for staining are listed, * - indicates false colored images to match markers shown here with Figure 7. Scale bars = 200 μm.
